# Extracellular Bacterial Production of DNA Hydrogels–Toward Engineered Living Materials

**DOI:** 10.1002/smll.202502199

**Published:** 2025-03-27

**Authors:** Philipp Gaspers, Christoph Bickmann, Christina Wallner, Daniel Baron Diaz, Dirk Holtmann, Johannes Gescher, Kersten S. Rabe, Christof M. Niemeyer

**Affiliations:** ^1^ Institute for Biological Interfaces 1 (IBG 1) Karlsruhe Institute of Technology (KIT) Hermann‐von‐Helmholtz‐Platz 1 76344 Eggenstein‐Leopoldshafen Germany; ^2^ Institute of Technical Microbiology University of Technology Hamburg (TUHH) Kasernenstraße 12 21073 Hamburg Germany; ^3^ Institute of Process Engineering in Life Sciences 2 – Electrobiotechnology Karlsruhe Institute of Technology (KIT) Fritz‐Haber‐Weg 4 76131 Karlsruhe Germany

**Keywords:** BES, biofilms, DNA materials, engineered living materials, *S. oneidensis*

## Abstract

Engineered Living Materials (ELMs) combine synthetic biology with artificial materials to create biohybrid living systems capable of replicating, self‐repairing, and responding to external stimuli. Due to their self‐optimization abilities, these systems hold great potential for biotechnological applications. This study is a first step toward ELMs based on DNA hydrogels, focusing on the production of biohybrid materials using the exoelectrogenic bacterium *Shewanella oneidensis*. To equip the bacterium with the functionality needed for building DNA hydrogels, inducible cell surface anchors are developed, which can bind exogenous polymerase via the SpyCatcher/SpyTag (SC/ST) technology. The process parameters for in situ production of DNA hydrogels are established, enabling the development of these materials in the context of living bacteria for the first time. Using an extracellular nuclease‐deficient *S. oneidensis* strain, stable biohybrid biofilms are generated directly on the surface of bioelectrochemical systems, showing the current generation. Given the high programmability and functionalization potential of DNA hydrogels, it is believed that this study represents a significant step toward establishing dynamic biohybrid material systems that exhibit both conductivity and metabolic activity.

## Graphical abstract

The polymerase phi29‐ST, immobilized via SpyCatcher surface anchors on the exoelectrogenic bacterium S. oneidensis, enables the formation of DNA hydrogel materials in direct proximity to living cells. This results in a robust biofilm that enhances the current generation. Since the formation and degradation of the biofilm and DNA material can be controlled through polymerization or nuclease digestion, this approach opens the door to new types of Engineered Living Materials (ELM).

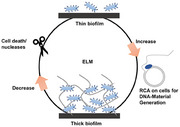

## 1 Introduction

The use of biological materials and mechanisms is the key to new technological applications for a resource‐conserving, sustainable economy. Nature impressively demonstrates that systems of incredible complexity can be created by a constant flow of small amounts of energy and matter. This dissipative process of “self‐organization” enables maximum programmable replication, repair, regulation, and environmental responsiveness with minimal energy consumption. Despite their enormous potential, the implementation of self‐organization processes for technical production routines remains largely untapped.^[^ ^1^, ^2^, ^3^, ^4^ ^]^ In order to develop the principle of biological self‐organization for technical applications, artificial dissipative systems are being intensively investigated today and the novel research field of “Engineered Living Materials” (ELMs) has been developed in recent years.^[^ ^5^, ^6^ ^]^ It seeks to develop living material systems that combine the dynamic properties of biological systems in terms of self‐regeneration and environmental sustainability with the robustness of traditional synthetic materials through the interdisciplinary combination of materials science and synthetic biology.

The development of artificial dissipative systems based on self‐organization is still in its early stages, with few applications emerging, primarily targeting protein‐ and peptide‐based materials for biomedical applications.^[^ ^5^, ^6^, ^7^, ^8^ ^]^ Although these promising early applications primarily address biomedical challenges associated with eukaryotic cells—offering tailored functionalities such as integration with host tissues, controlled drug release, and self‐healing, microbial organisms also hold significant potential for developing ELMs to enhance biotechnological processes.^[^ ^9^ ^]^ Several potential exoelectrogenic microorganisms have been identified,^[^ ^10^, ^11^ ^]^ with the well‐investigated exoelectrogenic bacterium *Shewanella oneidensis* (*S. oneidensis)* MR‐1^[^ ^12^, ^13^ ^]^ being frequently used for biotechnological applications, such as wastewater treatment, bioremediation, as well as the production of valuable metabolites generating electricity or hydrogen as a by‐product.^[^ ^14^, ^15^ ^]^ This facultative anaerobic bacterium is capable of reducing insoluble electron acceptors such as metals or electrodes conducting extracellular electron transfer.^[^ ^16^ ^]^ To this end, *S. oneidensis* possesses a sophisticated system of multi‐heme c‐type cytochromes across different compartments of the cell. The outer membrane cytochromes MtrC, MtrF, and OmcA allow direct electron transfer from the cell surface to insoluble electron acceptors in direct proximity, while soluble redox molecules like flavines serve as cofactors for DET or solely as shuttles for mediated electron transfer over larger distances.^[^ ^17^, ^18^ ^]^ Since *Shewanella* species typically form only thin biofilms, the resulting current generation is limited. Consequently, several studies have focused on increasing biofilm thickness to boost current output, either by genetically modifying *S. oneidensis*, engineering the electrode, or using electrically active materials to create conductive biofilms.^[^ ^19^, ^20^, ^21^, ^22^, ^23^, ^24^, ^25^, ^26^ ^]^

Many of these methods show promise, however, they tend to produce static biofilm compositions that do not allow for targeted adjustments. This is a limitation since biofilm composition affects factors like diffusion, which could reduce current output, for instance, due to proton accumulation.^[^ ^27^ ^]^ Although DNA, as so‐called “extracellular DNA (eDNA)”, is a natural component of biofilms,^[^ ^28^ ^]^ artificial DNA materials have rarely been used to create conductive biofilms in bioelectrochemical systems. Furthermore, fundamental concepts of ELM development have not yet been applied to DNA‐based materials systems. The goal of the present work was to explore the first steps toward ELMs based on DNA materials, focusing on the production of biohybrid materials using the exoelectrogenic bacterium *S. oneidensis*. DNA hydrogels, known for their versatility, biocompatibility, stimuli‐responsiveness, and programmable structure, have emerged in recent years as promising candidates for diverse applications such as drug delivery, tissue engineering, and biosensing,^[^ ^29^, ^30^, ^31^, ^32^ ^]^ but their use for cultivation of microbial cells has been little studied.^[^ ^33^ ^]^

The strategy of our work was to combine DNA hydrogels with living *S. oneidensis* cells to create biohybrid, synergistically interacting composite materials. In this approach, the DNA material would support the formation of microbial biofilms, while the microbial cells could build and/or degrade the DNA material to achieve optimal growth conditions. The advantage of bacteria producing the DNA material themselves, rather than using pre‐polymerized DNA or another polymer, is that in ELMs, living cells act as material factories, dynamically regulating material synthesis and degradation to create synergistic effects. This allows the production of only the material needed for optimal growth and power generation at any given time. Hence, such hybrid materials could exhibit the general properties of the microbial system, i.e., autonomous growth, metabolism, and response to the environment, and on the other hand, their physical properties (morphology, viscosity, conductivity) could be utilized to improve the electrode activity and simultaneously the volumetric productivity of a biotechnological process catalyzed by the microbes within a bioelectrochemical system (BES).

**Figure 1:**
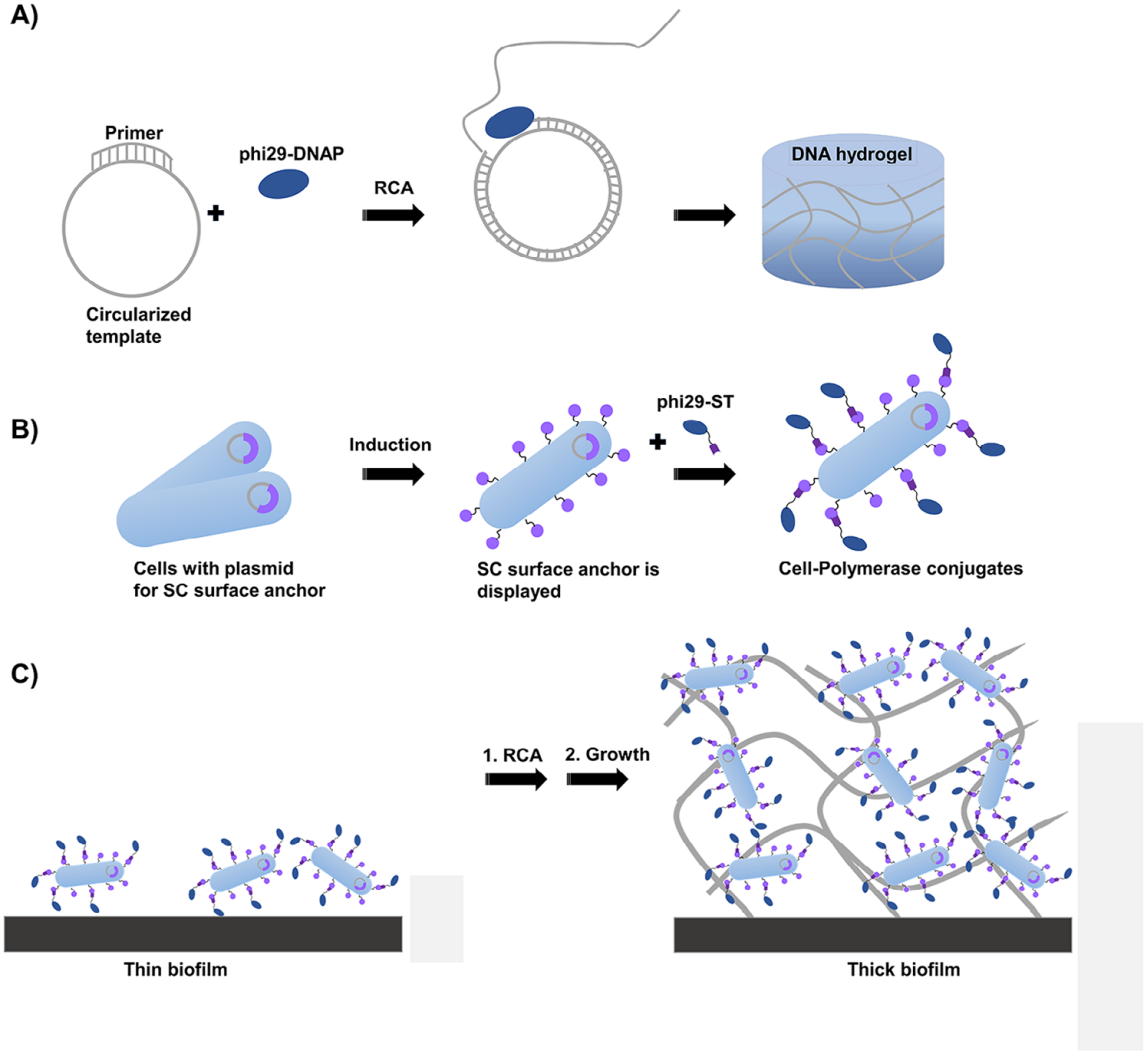
Schematics of the ELM approach developed in this study for enhanced growth of conductive S. oneidensis biofilms. A) phi29‐DNAP is capable of producing DNA hydrogels. B) S. oneidensis strain harboring a plasmid for the presentation of SC surface anchor upon an external stimulus to enable the immobilization of the phi29‐DNAP tagged with the corresponding binding partner. C) S. oneidensis does not create thick biofilms under anaerobic conditions. Cells externally coupled with phi29‐DNAP are being investigated for whether they can produce DNA hydrogels on demand, which could enhance the thickness of biofilm and the performance of a bioelectrochemical system by generating conductive biofilms. Note that the high programmability and functionalizability of DNA hydrogels could be leveraged to efficiently and easily immobilize various redox‐active molecules or conductive materials in close proximity to bacterial cells, thereby creating opportunities to further enhance the power generation of the biohybrid system.

While degradation can easily occur through bacterial secretion of nucleases, the phi29 DNA polymerase (phi29‐DNAP) required for DNA hydrogel synthesis cannot yet be produced by *S. oneidensis*. DNA hydrogels are synthesized using rolling circle amplification (RCA), in which a circular template is extended by phi29‐DNAP after hybridization with an oligonucleotide primer to generate long concatemeric stretches of single‐stranded DNA up to 20000 nucleotides in length, which ultimately form viscous DNA hydrogels due to physical entanglement (**Figure** **1**A). To enable *S. oneidensis* to produce DNA hydrogels, the phi29‐DNAP would need to be displayed on its surface. Due to the challenges of directly expressing and presenting the bulky phi29‐DNAP (molecular weight: 67 kDa) on the surface of *S. oneidensis*, we present an alternative approach focused on immobilizing phi29‐DNAP using SpyTag/SpyCatcher (ST/SC) technology.^[^ ^34^ ^]^ This coupling system allows for the rapid and irreversible conjugation of recombinant proteins and has recently been adapted to enable the display of complex proteins on the surface of cells.^[^ ^35^, ^36^ ^]^ To this end, we have generated novel *S. oneidensis* strains that utilize the outer membrane protein MtrF to present SC molecules on the cell surface for coupling with ST‐fused phi29‐DNAP (Figure 1B). Furthermore, since *S. oneidensis* is known to secret nucleases^[^ ^37^, ^38^ ^]^ which might interfere with DNA hydrogel generation, an *S. oneidensis* strain deficient for the nucleases EndA, ExeM, and ExeS was constructed.^[^ ^37^, ^38^ ^]^ This strain was then compared with wild‐type *S. oneidensis* strains in terms of the ability to generate DNA hydrogels and produce thicker biofilms and enhanced electrical current in bioelectrochemical systems (Figure 1C).

## 2 Results and Discussion

### 2.1 Generation of an Extracellular Nuclease Deficient *S. oneidensis* Strain

Based on the literature, the genes *exeS* (SO_1844), *endA* (SO_0833), and *exeM* (SO_1066) were identified as encoding for extracellular nucleases, which might enable *S. oneidensis* to digest extracellular DNA such as the hydrogel to be produced by the phi29‐DNAP.^[^ ^37^, ^38^ ^]^ To remove these genes from the genome of *S. oneidensis* the regions 500 bp upstream and downstream of the respective genes were integrated into the suicide vector pMQ150^[^ ^39^ ^]^ via PCR (For details on primer sequences see Table S1, Supporting Information), the vector transformed into *E. coli* and transferred into *S. oneidensis* via conjugation, resulting in a triple deletion strain, further referred to as ∆N.

### 2.2 Presentation of Surface Anchored‐SC/ST‐DNAP on *S. oneidensis*

**Figure 2:**
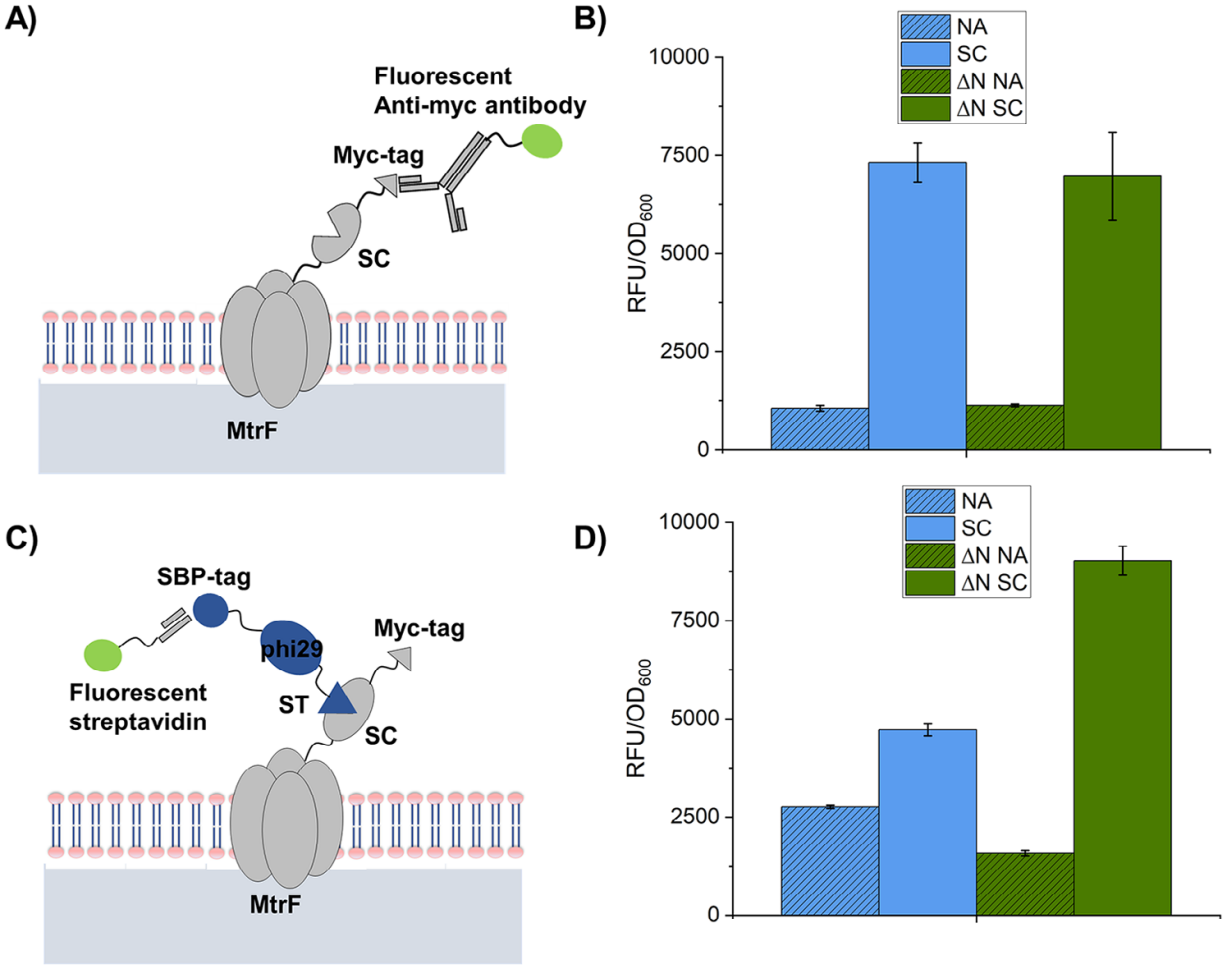
Evaluation of surface presentation of SC anchors on S. oneidensis. A) Schematic of the enzyme‐to‐cell coupling system using SC fused to the membrane protein MtrF of S. oneidensis. The surface presentation can be verified directly by using fluorescent antibodies targeting the Myc‐tag which is located at the C‐terminus of the MtrF‐SC fusion protein. B) Measured fluorescence of SC‐expressing strains incubated with anti‐Myc^DL488^. Strains without plasmid for surface anchor expression (NA) served as negative control. C) Schematic illustration of phi29‐ST coupling to SC‐presenting cells and subsequent verification with fluorescent streptavidin. D) Measured fluorescence of cells incubated with phi29‐ST and STV^AF488^. Respective microscopic images of the samples are shown in Figure S1 (Supporting Information).

To allow for immobilization of phi29‐DNAP on cell surfaces we generated *S. oneidensis* strains that present suitable surface anchors upon an external trigger. *S. oneidensis* harbors the protein MtrF involved in the respiration of insoluble electron acceptors. This c‐type cytochrome is located in the outer membrane and therefore a promising fusion partner to present SC surface anchors for immobilization of large proteins. Consequently, we introduced the SC‐tag at the C‐terminus of the MtrF sequence located on a pBAD202 plasmid together with a Myc‐tag to enable detection via immunofluorescence staining (Tables S2,S3, Supporting Information).^[^ ^36^, ^40^ ^]^ The resulting vector was transformed using wild‐type and nuclease‐deficient *S. oneidensis* resulting in the novel strains dubbed SC and ∆N SC, respectively (Table S4, Supporting Information). For initial characterization, cells were incubated anaerobically in an M4 medium and subsequently induced for expression of the surface anchor by adding arabinose. Following night growth and harvesting, cells were stained using DyLight488 conjugated anti‐Myc antibody (anti‐Myc^DL488^),^[^ ^35^ ^]^ and, after washing the cells thoroughly, fluorescence intensity was measured using a fluorescence plate reader (**Figure** **2**A,B). Cells harboring the empty pBAD plasmid and therefore expressing no anchor (NA) served as control (further denoted as NA and ∆N NA). The strains in which the SC surface anchor was overexpressed showed a significantly (≈5‐fold) higher fluorescence than both NA strains that contained the empty pBAD vector. Additional experiments using fluorescence microscopy (Figure S1A–D, Supporting Information) confirmed these results and showed that surface display of SC anchors using the *S. oneidensis* membrane protein MtrF as a fusion partner was successful.

Next, the binding functionality of the surface‐presented SC anchor was investigated by adding the complementary ST‐modified variant phi29‐ST which also contains a streptavidin‐binding tag (SBP)^[^ ^41^ ^]^ to the *S. oneidensis* cells (Figure 2C,D). After adding AlexaFluor488‐labelled streptavidin (STV^AF488^), cells were washed thoroughly and fluorescence was measured with a fluorescence plate reader. The results revealed significantly higher fluorescence signals for cells expressing the anchor as compared to control samples with cells harboring the empty vector. Additional microscopy analyses (Figure S1, Supporting Information) showed that a small portion of NA cells was fluorescent, suggesting that unspecific adsorption of phi29‐DNAP and/or STV^AF488^ occurs on the cells. However, the significant difference in the measured fluorescence — by a factor of 2 and 5.5 for the SC and ∆N SC strains, respectively, compared to the control strains — clearly showed that a specific binding occurs between phi29‐ST and SC‐presenting cells.

### 2.3 Determining Suitable Conditions for Combining RCA with the Simultaneous Cultivation of *S. oneidensis*

**Figure 3:**
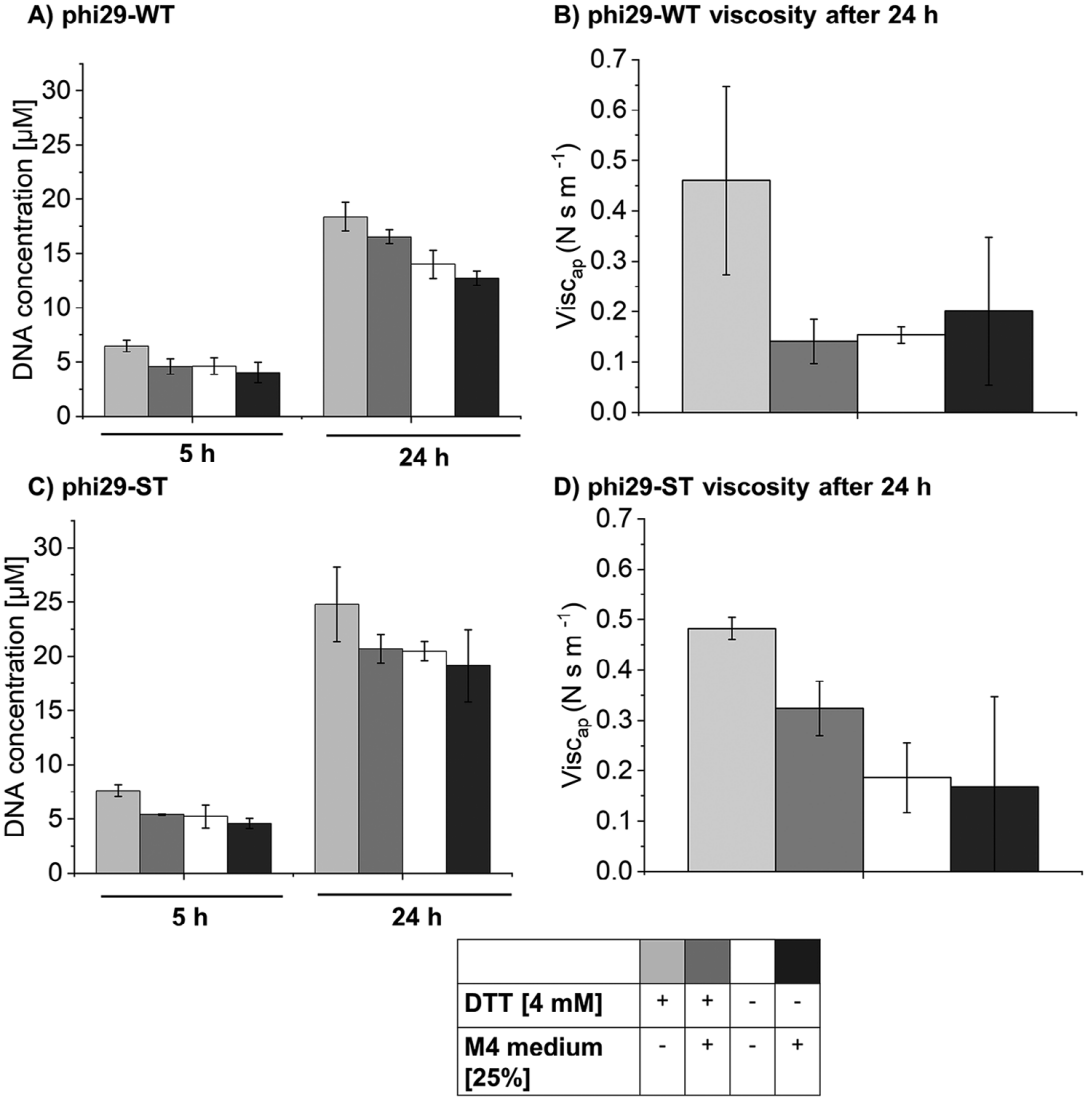
Characterization of the RCA process performed by phi29‐WT and phi29‐ST under variable reaction conditions by adding 25% M4 medium in the presence and absence of 4 mM DTT (standard conditions). A,C) Determination of the absolute amount of DNA produced in the RCA reaction mixtures at different time points by qPCR. B,D) Determination of the apparent viscosity of the DNA hydrogels produced after 24 h of reaction time by micromechanical penetration measurements. For a representative calibration curve of qPCR see Figure S8 (Supporting Information).

After successfully verifying the surface presentation of MtrF‐SC fusion proteins, we identified the conditions that enable the simultaneous cultivation of *S. oneidensis* and the execution of RCA for the intended production of DNA hydrogel with phi29‐DNAP in the presence of living *S. oneidensis*, referred to hereafter as “in situ RCA”. Initially, the influence of different ratios of M4 medium and phi29 reaction buffer (0/100, 25/75, 50/50, and 100/0) on the growth of *S. oneidensis* strains SC and ∆N SC at the optimal growth temperature of 30 °C was examined. To also account for the potential impact of the reducing agent DTT, which is part of the phi29 reaction buffer, cultures in the presence and absence of DTT were also investigated (Figure S2, Supporting Information). The results demonstrated that the *S. oneidensis* strains, as expected, showed optimal growth in 100% M4 medium. DTT appeared to slightly inhibit the growth of both strains, while incubation in 100% phi29 reaction buffer resulted in static behavior. However, cells from all tested conditions were able to resume growth in fresh M4 medium after a brief initial lag phase (Figure S3, Supporting Information), indicating that none of the conditions were unsuitable for further experimentation. Since cells were able to grow in mixtures of 25% M4 medium and 75% phi29 reaction buffer, we next investigated whether phi29‐DNAP is capable of performing RCA under these conditions using the circular single‐stranded template C (for details on secondary structure and electrophoretic characterization, see Figures S4,S5, Supporting Information). Initial investigations were conducted using a fluorescence assay based on the intercalating dye SYBR Green I. The results suggested that the addition of 25% M4 medium to the RCA buffer significantly impaired DNAP activity, especially when no DTT was present in the reaction (Figures S6,S7, Supporting Information). However, as this assay can be affected by artifacts, we decided to quantify the absolute amount of DNA hydrogel produced using a qPCR protocol^[^ ^42^ ^]^ and to assess the mechanical properties through micromechanical indentation analysis^[^ ^43^ ^]^ (**Figure** **3**).

**Figure 4:**
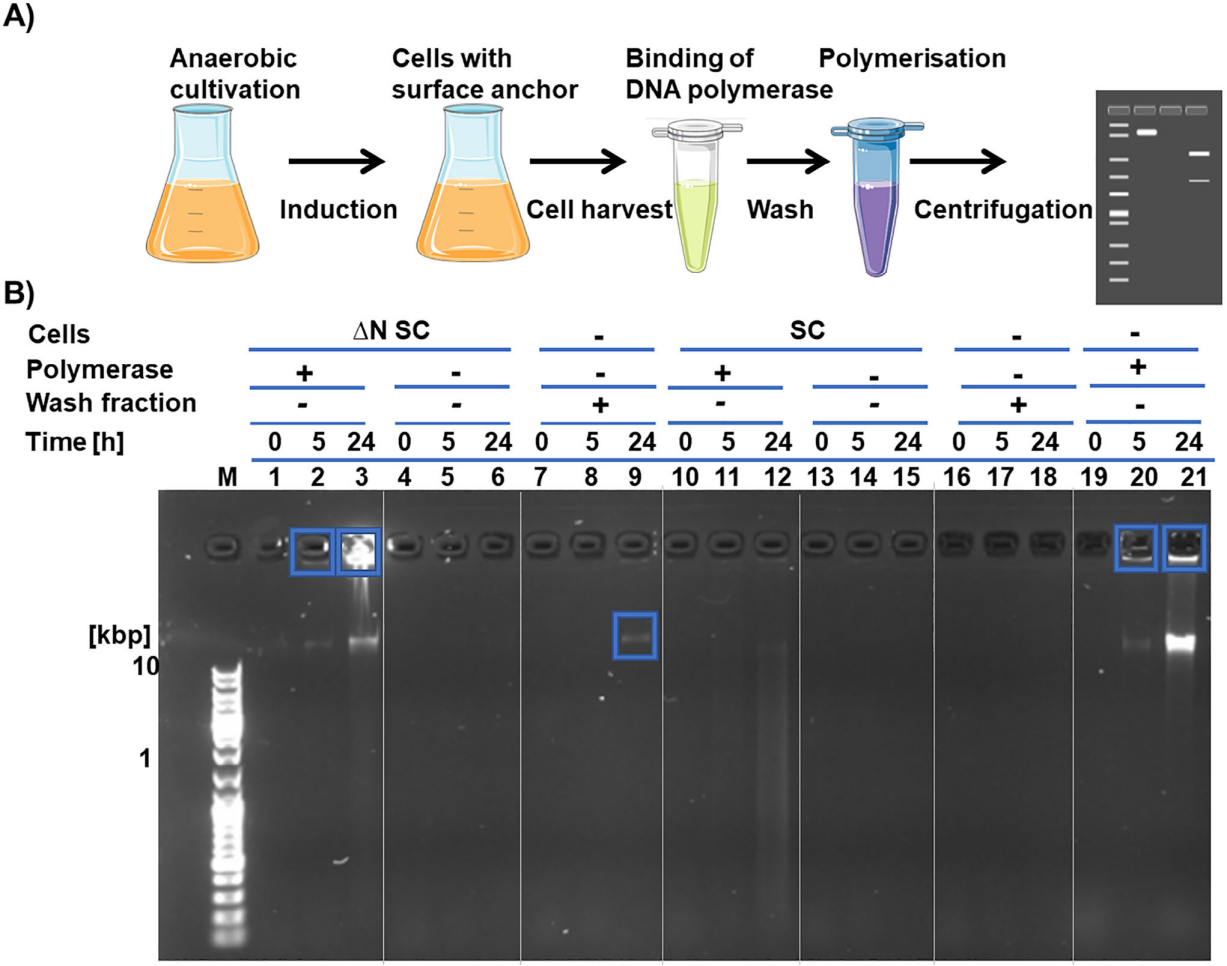
In situ, RCA mediated by phi29‐ST immobilized on ∆N SC and SC strains. A) Cells harboring the plasmid for MtrF‐SC expression were grown under anaerobic conditions and induced for expression of the SC surface anchor. Cells were then harvested and loaded with phi29‐ST polymerase. After washing the cell suspension thoroughly, cells were added to the RCA standard reaction mix. Samples were taken at certain time points and analyzed by agarose gel electrophoresis. B) 1% agarose gel analysis of RCA mixture incubated with S. oneidensis strains ∆N SC and SC. Lanes 1–3 and 10–12: ∆N SC and SC cells coupled with polymerase. Lanes 4–6 and 13–15: ∆N SC and SC cells not coupled with polymerase. Lanes 7–9 and 16–18: Cell‐free supernatant of the last wash step of cells coupled with polymerase. 19–21: positive control using free phi29‐ST polymerase for RCA. Blue boxes indicate RCA products.

The results of the quantitative analyses showed that DNA yields using RCA under standard conditions (bright grey bars) were highest for both the wild‐type phi29‐DNAP (phi29‐WT) and the phi29‐ST variant to be used for cell surface coupling (Figure 3A,C). In terms of mechanical properties, RCA with both phi29‐DNAP variants under standard conditions produced DNA hydrogels with viscosities that were 2–3 times higher than those of the gels produced under varied reaction conditions (Figure 3B,D). Since *S. oneidensis* was able to survive for up to 24 h in 100% phi29 reaction buffer with 4 mM DTT (standard RCA conditions) (Figures S2,S3, Supporting Information), these conditions were selected for further experiments aimed at producing DNA hydrogels by in situ RCA with phi29‐ST immobilized on the cell surface (**Figure** **4**).

### 2.4 In Situ Formation of DNA Hydrogels with DNAP Immobilized on *S. oneidensis*

Following the successful preliminary work, the strains SC and ∆N SC were used for the in situ polymerization of DNA hydrogels in the RCA reaction mix containing no M4 medium. A schematic of the procedure is shown in Figure 4A. Analysis by agarose gel electrophoresis (Figure 4B) showed the characteristic high molecular weight band of DNA hydrogel for reactions carried out with strain ∆N SC that was loaded with phi29‐ST. The band was visible after reaction times of only 5 h and increased strongly until the end of the experiment at 24 h (lanes 2, and 3, respectively). In contrast, no such bands were visible in the control samples where no polymerase was added to the cells (lanes 4–6), indicating that *S. oneidensis* does not possess its own polymerases that can produce high molecular weight DNA products. Lanes 7–9 show samples in which the RCA mix was prepared with the cell‐free supernatant from the last wash of cells incubated with polymerase. The absence of DNA‐hydrogel bands indicates that there were no unbound polymerases and thus phi29‐ST was indeed firmly immobilized on ∆N SC, capable of performing in situ RCA. Interestingly, for the in situ RCA performed with strain SC (Lane 12), only one smear was observed in the sample where polymerase was added to the cells. This finding is consistent with the secretion of nucleases by wild‐type *S. oneidensis*, as reported in the literature,^[^ ^37^, ^38^ ^]^ which here prevents the efficient formation of higher molecular weight products by enzymatic degradation.

These results were confirmed by further investigations into the influence of different strains on DNA hydrogel stability. For this purpose, DNA hydrogels labeled with Cy5‐dUTP for fluorescence marking were produced in microplate cavities, making them analyzable by confocal fluorescence microscopy (Figure S9A, Supporting Information). Subsequent addition and incubation with the SC strain led to the complete degradation of the DNA hydrogel, while samples incubated with ∆N SC showed a well‐preserved and intact hydrogel. Analysis of the samples using agarose electrophoresis confirmed these findings, as no high‐molecular‐weight DNA bands were detectable for the wild‐type strain after 24 h (Figure S9B, Supporting Information).

**Figure 5:**
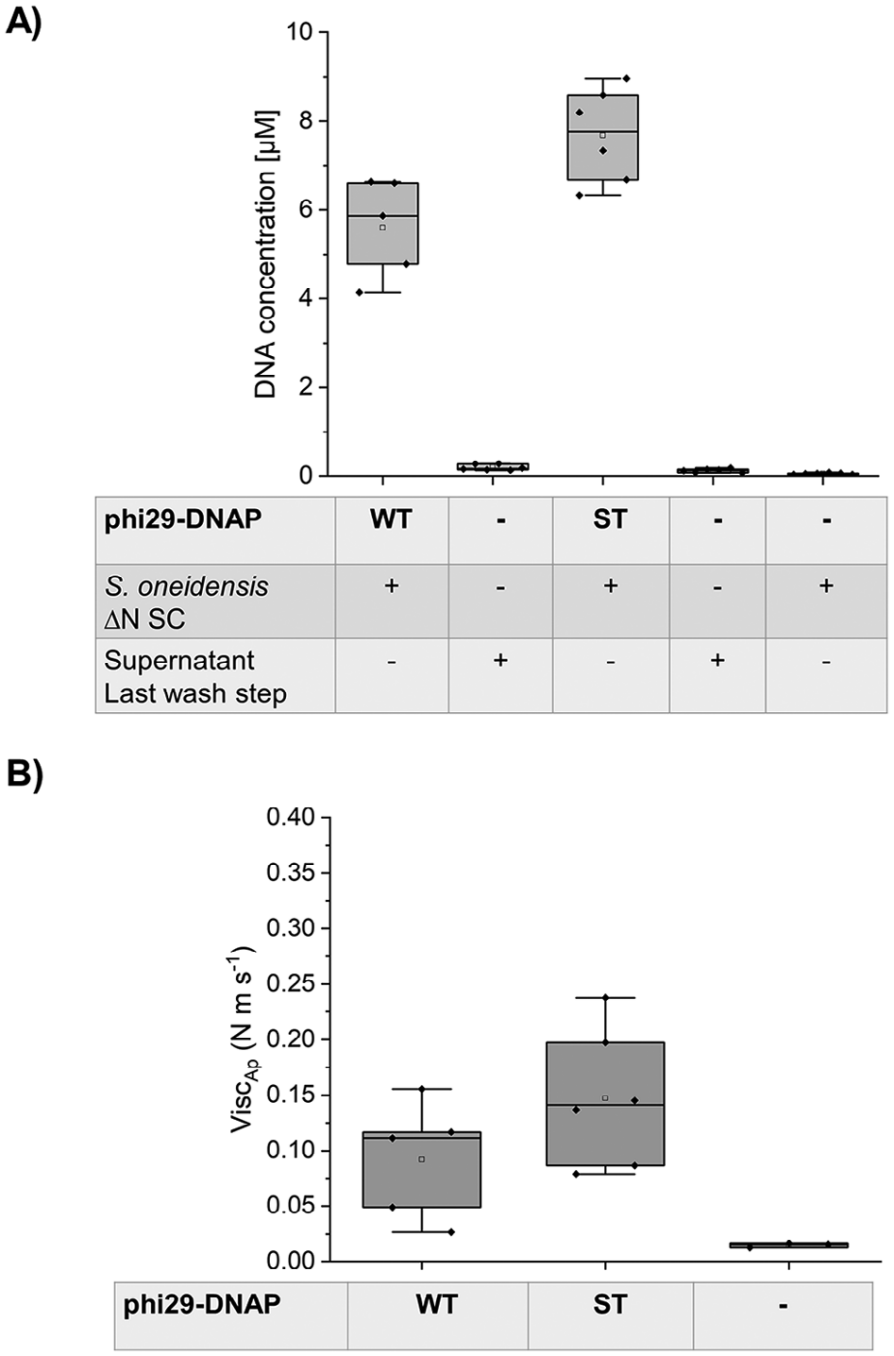
Characterization of DNA hydrogel generated by phi29‐DNAP variants immobilized on S. oneidensis strain ∆N SC. Cells incubated with phi29‐ST were used to perform in situ RCA. Cells incubated with phi29‐WT and cells with no added polymerase served as controls. A) RCA products generated after 24 h were quantified by qPCR. B) Micromechanical indentation analysis of the mechanical properties of DNA hydrogels prepared with ∆N SC cells incubated with phi29‐ST, phi29‐WT, or no polymerase. Note that cells incubated with phi29‐WT produced DNA and viscoelastic material, suggesting some nonspecific polymerase binding. However, the specific interaction between phi29‐ST and the SC surface anchor led to higher hydrogel production and viscosity, confirming that ST/SC coupling enhances RCA productivity.

To confirm the generation of DNA hydrogels by cell‐immobilized phi29‐ST, we performed in situ RCA using the ∆N SC strain, followed by qPCR and micromechanical indentation analysis (**Figure** **5**). As controls, we used cells without polymerase, as well as the supernatant from the final wash step to confirm the absence of unbound polymerase. Additionally, we included phi29‐WT, which does not bind to the cells, as a further control. The results (Figure 5A) revealed that cells incubated with phi29‐ST polymerase produced ≈8 µm of RCA product, exceeding the amount generated by cells incubated with phi29‐WT by ≈30%. Samples without added polymerase, as well as the supernatant from the last washing step, showed no detectable DNA production, confirming that the addition of polymerase is essential for in situ RCA. Consistent with the qPCR data, samples with ∆N SC cells incubated with phi29‐ST exhibited the highest apparent viscosity, whereas cells without polymerase showed no measurable viscosity (Figure 5B). Cells incubated with phi29‐WT also generated a viscoelastic material, though with significantly lower viscosity. These findings confirmed that neither host polymerase nor unbound polymerase contributed to DNA synthesis and that the DNA hydrogel was exclusively produced by phi29‐DNAP immobilized on the cell surface. Of note, for cells incubated with phi29‐WT, DNA generation, and viscoelastic material formation were observed, suggesting some level of nonspecific polymerase binding to the cells.

However, the specific interaction between phi29‐ST and the SC surface anchor resulted in greater hydrogel production and higher viscosity, demonstrating that direct coupling via the ST/SC system positively influenced RCA productivity. These results confirmed that viscoelastic DNA hydrogels can be produced in situ using phi29‐ST presented on ∆N SC cell surfaces, making this approach potentially suitable for generating DNA materials in microfluidic BES systems. Furthermore, since the wild‐type strain degrades the material completely within 24 h, preventing the formation of new DNA, these findings underlined the necessity of using the nuclease‐deficient *S. oneidensis* strain ∆N SC for successful DNA material synthesis via in situ RCA as the foundation for a self‐optimizing ELM system.

### 2.5 Generation of DNA Hydrogels Inside the Microfluidic Bioelectrochemical System

**Figure 6:**
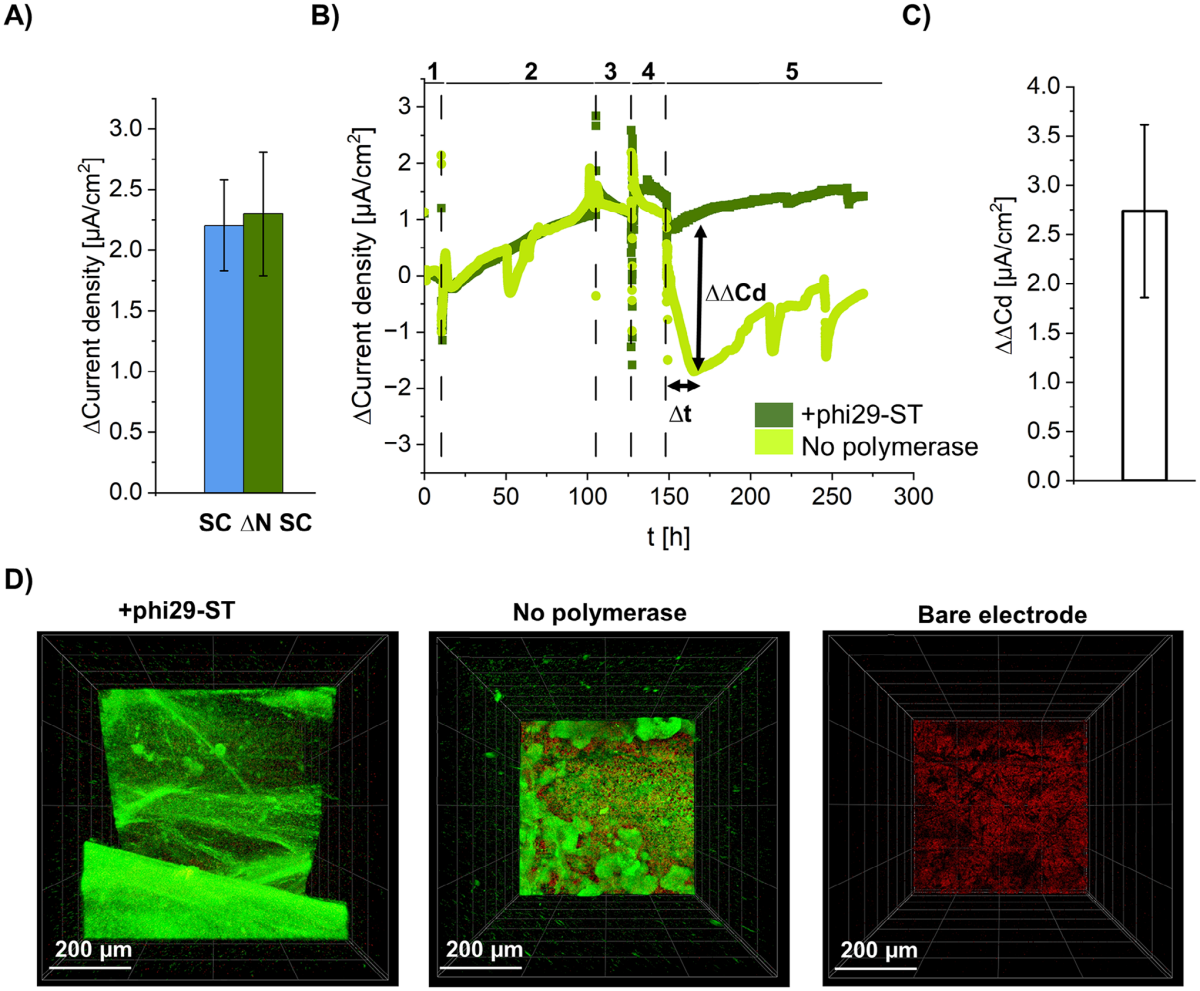
A) Achieved current density of different S. oneidensis strains SC and ∆N SC on bare graphite electrodes at a potential of −199 mV vs Ag/AgCl. B) Course of current density for two exemplary BES chips through different stages of cultivation and production of DNA hydrogel. 1: Equilibration of the system under abiotic conditions; 2: Growth of S. oneidensis ∆N SC on bare graphite electrode in M4 chip medium; 3: Induction of MtrF‐SC surface anchor expression; 4: Addition of RCA reaction mixture containing (green) or lacking (bright green) phi29‐ST to the cells inside the BES. Medium flow was stopped for 24 h, 5: Subsequent growth of S. oneidensis ∆N SC after flow change to M4 chip medium. C) Bar chart representation of the difference in current density between three independent BES chips where polymerization was conducted and two independent control chips without polymerization. The difference was calculated by extracting the current density 16 h after polymerization (see B), phase 4, (∆t)) for both sets of chips and then subtracting the values of the control chips from those of the chips where polymerase was added, resulting in ∆∆Current density (see B), ∆∆Cd). D) Microscopy images of the BES‐chips after experiment shown in (B). SYBR Green I stained cells and the electrode surface (see bare electrode for comparison, right) are shown in green and red colors, respectively, as observed on the surface of chips treated with polymerase (left) or no polymerase (middle).

To characterize the performance of novel strains and conduct in situ polymerization experiments, a recently developed microfluidic bioelectrochemical system (BES) was used.^[^ ^44^ ^]^ For photographic images, see Figure S10 (Supporting Information). To initially investigate whether the knockout of the three nucleases ExeM, ExeS, and EndA in the *S. oneidensis* strain ∆N SC affected its ability to generate current, chronoamperometric experiments were performed with strains ∆N SC and SC for 150 h at a potential of −199 mV vs Ag/AgCl in the microfluidic bioelectrochemical system (**Figure** **6**A). The results showed that the current density for the nuclease‐deficient strain ∆N SC is similar to that of the SC strain, making it suitable for further investigations.

We then carried out experiments with the aim of generating DNA hydrogels in situ with phi29‐ST immobilized on cells inside the microfluidic BES containing two measurement chips (green and bright green curves, in Figure 6B). To this end, the *S. oneidensis* ∆N SC strain was first cultivated for 75 h at a potential of −199 mV vs Ag/AgCl (Figure 6B, point 2). The chronoamperometric signals showed a linear increase in current density related to the growth of *S. oneidensis*. Subsequently, expression of the MtrF‐SC surface anchor was induced by adding arabinose to the flow medium (Figure 6B, point 3). Both operations led to short‐term fluctuations in the electrochemical read‐out, but the overall current density remained at the same level. To minimize the number of manual interventions in the highly sensitive BES, the polymerization was then initiated directly by injecting 1000 µL of RCA mixture with polymerase into the BES while the medium flow was stopped (Figure 6B, point 4). For this, phi29‐ST was added to the RCA mix (green curve), and as a control, no polymerase was added (bright green curve). After the injection of the polymerization mixture, the signal measured by the chip to which no polymerase was added dropped continuously and only increased again after a recovery time of ≈2 days (bright green in Figure 6B, phase 5). The latter might be due to biofilm detachment due to a change in medium/buffer conditions. In contrast, the signal remained approximately constant for the chip with polymerase and continued to increase slowly until the end of the experiment (green curve). Constant current production might be due to biofilm stabilization based on the produced eDNA network (Figure 6D). Repetitions of the experiment yielded similar results in the course of the current density of BES chips with phi29‐ST in the RCA solution compared to the control sample without polymerase. To visualize the positive influence of polymerization on electrode surfaces and current production, we calculated the difference in current density (∆∆Current density, ∆∆Cd) between chips treated with phi29‐ST and control chips, displaying it as a bar graph (Figure 6C). For this purpose, we initially selected the current values of each chip 1.5 h after the end of polymerization (equals ≈10 volumes of medium exchange in the chips) as the reference value to reduce any measurement artifacts from the conversion from RCA conditions to the M4 chip medium. Based on this reference, we determined the current density of the individual chips after a further 15 h (∆t) and calculated the ∆∆Cd by subtracting the current density of control chips from that of the chips where polymerase was added to the RCA mix which resulted in a value of 2.7 ± 0.9 µA cm^−2^. Thus, the electrochemical read‐out provided the first evidence for the formation of DNA hydrogels and their positive effect on the growth of *S. oneidensis* cells.

To verify that a DNA hydrogel had indeed been generated, the BES chips were removed from the cell and stained with SYBR Green I. Microscopic imaging (Figure 6D) clearly showed that the chip to which polymerase was added had a pronounced biofilm (green) covering the entire graphite electrode (red), whereas only a thin and discontinuous cell layer was observed on the control chip. This indicated that the polymerization process did indeed have a positive influence on the thickness of the biofilm. In summary, the results demonstrated that it is possible to perform hydrogel synthesis with *S. oneidensis* on electrode surfaces over extended periods of up to 24 h without negatively impacting the cells.

Following the successful development of protocols for hydrogel synthesis in microfluidic BES, we aimed to explore whether these protocols could also be applied to standard batch‐operated BES, which are predominantly used in bioelectrochemistry. For these experiments, we utilized the recently developed E‐cuvette system,^[^ ^45^, ^46^ ^]^ consisting of screen‐printed electrodes integrated into commercial cuvettes, enabling parallelized measurements of up to eight independent BES (Figure S11, Supporting Information). In this system, we analyzed the in situ polymerization capabilities of the wild‐type strain SC and the nuclease‐deficient strain ∆N SC, comparing the maximum current generated before and after polymerization. The results, shown in Figure S12 (Supporting Information), clearly demonstrated that polymerization led to a significantly increased current production for the nuclease‐deficient strain ∆N SC, whereas only minor increases were observed for the wild‐type strain. Microscopic analysis of the electrode surfaces after the experiment confirmed enhanced biofilm formation exclusively for the ∆N SC strain. Collectively, these findings verify that this strain is essential for material formation and superior to the wild‐type *S. oneidensis* in terms of current production.

## 3 Conclusion

This study aimed to explore a new approach for the production of biohybrid material systems, which could lead to the development of engineered living materials. We successfully developed new *S. oneidensis* strains capable of presenting the SpyCatcher protein on their surface. This first‐time demonstrated functionalization allows the specific biomolecular coupling of bulky enzymes, such as phi29‐DNAP, in close proximity to the cell, which can be used for the formation of DNA hydrogels by in situ RCA. To the best of our knowledge, this also is the first study demonstrating the synthesis of these widely applicable, molecularly programmable materials in a microbial context. The nuclease‐deficient *S. oneidensis* strain used here significantly enhanced the stability of DNA hydrogels compared to the wild‐type strain and generated similar current levels inside bioelectrochemical systems (BES). Polymerization experiments conducted directly within the BES suggest that the combination of the nuclease‐deficient *S. oneidensis* strain with SC surface anchors represents a viable platform for the dynamic production of DNA hydrogels on electrode surfaces. Further experiments are required to precisely characterize the amount of DNA produced and the viscoelastic properties of the DNA hydrogel formed in situ in the BES. Additionally, systematic electrochemical studies are needed to investigate how the in situ‐generated DNA hydrogels affect the growth and performance of *S. oneidensis* and whether the organism's growth and performance on the electrodes could potentially be enhanced by integrating conductive materials or redox‐active mediators into the DNA hydrogel (see note in Figure 1). Future research should also investigate the effect of the cultivation of *S. oneidensis* in DNA hydrogels on its metabolism and how integration of, e.g., stimulus responsiveness of the DNA hydrogels could be utilized to improve the ELMs biocatalytic performance. In the long term, this approach could lead to the development of complex Es, where growth and degradation are controlled through the addition or secretion of DNAP and nucleases, leading to highly stable self‐regenerating material.

Furthermore, although the presented work focused on microbial systems, the strategies and protocols developed here could be adapted for use with eukaryotic cell systems. For instance, DNA‐hybrid materials can serve as cell‐instructive matrices for the cultivation and differentiation of stem cells^[^ ^47^, ^48^, ^49^ ^]^ or for protection against stress‐induced autophagy.^[^ ^50^ ^]^ However, the DNA materials used so far are often expensive and not suited for triggered, site‐specific material formation. Given that animal cells exhibit a wide variety of surface proteins that can be genetically engineered,^[^ ^51^ ^]^ our bacterial hydrogel synthesis approach could be adapted to animal cells by using a suitable membrane protein for display phi29‐DNAP, leveraging RCA's cost‐effective and cell‐friendly nature for spatiotemporal control of materials formation. This approach could enable studying how material properties influence cell growth and differentiation and, combined with electronics, might facilitate the creation of neural structures inside tailored DNA architectures to investigate electrical communication.^[^ ^52^ ^]^ While large‐scale production and long‐term effects of DNA hydrogels on cellular behavior still need to be investigated, we believe this study represents a significant step toward establishing dynamic biohybrid material systems that exhibit both conductivity and metabolic activity.

## Conflict of Interest

The authors declare no conflict of interest.

## Supporting information



Supporting Information

## Data Availability

The data that support the findings of this study are available from the corresponding author upon reasonable request.
