# Supplementary material for: Extracellular Bacterial Production of DNA Hydrogels–Toward Engineered Living Materials
Source: Small. 2025 Mar 27;21(19):2502199. doi: 10.1002/smll.202502199 (PMC12067159; doi:10.1002/smll.202502199)
Supplement: Supplementary file 1 — Supporting Information [file SMLL-21-2502199-s001.pdf]

NANO · MICRO
small

Supporting Information

for *Small*, DOI 10.1002/smll.202502199

Extracellular Bacterial Production of DNA Hydrogels–Toward Engineered Living Materials

*Philipp Gaspers, Christoph Bickmann, Christina Wallner, Daniel Baron Diaz, Dirk Holtmann, Johannes Gescher, Kersten S. Rabe and Christof M. Niemeyer**

Supporting Information

Extracellular Bacterial Production of DNA Hydrogels - Towards Engineered Living Materials

Philipp Gaspers,[a] *Christoph Bickmann,*[a] *Christina Wallner,*[b] *Daniel Baron Diaz,*[c] *Dirk Holtmann,*[c] *Johannes Gescher,*[b] *Kersten S. Rabe*[a] *and Christof M. Niemeyer**[a]

[a] Karlsruhe Institute of Technology (KIT), Institute for Biological Interfaces 1 (IBG 1), Hermann- von- Helmholtz-Platz 1, 76344 Eggenstein-Leopoldshafen, Germany

[b] University of Technology Hamburg TUHH, Institute of Technical Microbiology, Kasernenstraße 12, 21073 Hamburg, Germany

[c] Institute of Process Engineering in Life Sciences 2 - Electrobiotechnology Karlsruhe Institute of Technology (KIT), Fritz-Haber-Weg 4, 76131 Karlsruhe, Germany

Email: niemeyer@kit.edu (C.M.N.); Tel.: + 49-721-608-23000

Table of contents

Materials and Methods………………………………………………………………………. 3

Chemicals………………………………………………………………………………………3

Construction of a *S. oneidensis* strain deficient for extracellular nucleases……………..……3

Cloning of genetic constructs for the surface presentation of ST/SC anchors on *S. oneidensis*……………………………………………………………………………….....3

Transformation of *S. oneidensis*………………………………………………………………..4

Verification of surface presentation of SC anchor proteins on *S. oneidensis* by immunofluorescence staining……………………………………………………………………..4

Verification of coupling efficiency of phi29-ST to SC anchor proteins on *S. oneidensis*………5

Cell survival assay…………………………………………………………………………..…..5

Circularization of RCA-template………………………………………………………………..6

RCA standard conditions………………………………………………………………………..6

SYBR Green I (SG) based RCA assay…………………………………………………………..6

qPCR quantification of RCA product……………………………………………………………..6

Rheology measurements……………………………………………………………………..…7

Stability assay of DNA-Hydrogels in the presence of wild type *S. oneidensis* and nuclease deficient *S. oneidensis* strains……………………………………………………………………7

In situ rolling circle amplification with phi29-ST immobilized on *S. oneidensis* MtrF-SC….…8

Production of Ag/AgCl reference electrodes……………………………………………….…..……8

Production of PDMS chips for the microfluidic bioelectrochemical reactor………………..…...8

Preparation of the microfluidic bioelectrochemical system……………………………………....9

Cultivation of *S. oneidensis* in the microfluidic bioelectrochemical system……………...…...9

Cultivation of *S. oneidensis* in parallelized Standard-BES……………………………………..10

Supporting information figures

Figure S1: Evaluation of coupling functionality of phi29-ST to cells presenting SC surface anchors…………………………………………………………………………………………12

Figure S2: Growth behaviour of *S. oneidensis* strains SC and ∆N SC in medium containing different ratios phi29 reaction buffer………………………………………………………………13

Figure S3: Regrowth of *S. oneidensis* in 100% M4 medium after 24 h incubation in different conditions…………………………………………………………………………………………14

Figure S4: Secondary structure prediction of the circular DNA template C and its complementary amplicon product C'……………………………………………………………15

Figure S5: Characterization of circular template C after ligation and purification. 2 µM of circularized template purified template C was loaded onto 12% Urea-PAGE………………..15

Figure S6: Activity screen of different commercial phi29 concentrations with a fluorescence-based rolling circle activity assay……………………………………………………………………16

Figure S7: Activity screen of different phi29-DNAP variants in varying reaction conditions ……………………………………………………………………………………………………17

Figure S8: Representative calibration curve obtained by qPCR to quantify the concentration of RCA products…………………………………………………………………………………17

Figure S9: Stability investigations of pure DNA hydrogel in the presence of SC and nuclease deficient ∆N SC strains………………………………………………………………………..18

Figure S10: Overview of the used microfluidic BES-system……………………………..…...19

Figure S11: Overview over the e-cuvette system used for BES-batch experiments…….….20

Figure S12: Assessment of current density and DNA hydrogel formation morphology using *S. oneidensis* strains SC and ∆N SC in a batch-operated E-cuvette BES equipped with eight parallelized, independent screen-printed gold electrodes (Figure S11)………………………21

Supporting information tables

Table S1: Sequences of primers used for the deletion of the nucleases ExeS, ExeM and EndA…………………………………………………………………………………………..23

Table S2: Genetic constructs used in this work…………………………………………………..23

Table S3: Amino acid sequences of MtrF-fusion proteins used in this study……………………………………………………………………………………..24

Table S4: Strains used in this study……………………………………………………….25
Table S5: Sequences of primer used for this work……………………………………....25
Table S6: Sequences of templates and primer used for RCA and qPCR………….……….…26
References……………………………………………………………………………….26

Material and Methods

Chemicals

All chemicals in this study were purchased. Oligonucleotides were ordered from Biomers, commercial enzymes and buffers from New England Biolabs, nucleotides from Jena Bioscience and graphite electrodes from müller & rössner. Water was used purified from Milli-Q machine from Merck.

Construction of a *S. oneidensis* strain deficient for extracellular nucleases

The regions 500 bp upstream and downstream of the genes exeS (SO_1844), endA (SO_0833), and exeM (SO_1066) were amplified using polymerase chain reaction (PCR).[37, 38] A detailed list of primers used in this study can be found in Table S1, Supporting Information. The resulting upstream and downstream regions were inserted into the BamHI and SalI (New England Biolabs) digested suicide vector pMQ150[39] via an isothermal assembly method[53] and subsequently transformed into E. coli WM3064. This construct was then conjugated into a *S. oneidensis* strain deficient in the λ-prophage.[54] Transconjugants of *S. oneidensis* Δλ containing the integrated mutagenesis vector were selected on Luria Bertani (LB) agar plates supplemented with kanamycin (50 µg/mL). Colonies were grown in the absence of antibiotics and subcultured twice in LB medium. Following overnight growth at 30°C, dilutions were plated on LB agar plates containing 10% sucrose and incubated overnight at 30°C. Plates were then replica-printed onto both LB plates and LB plates supplemented with kanamycin (50 µg/mL). Kanamycin-sensitive colonies were screened by PCR to identify strains with the respective deletions. The procedure was repeated until a strain with deletion of all three genes was acquired. The deletions were verified through commercial sequencing (Eurofins Genomics)

Cloning of genetic constructs for the surface presentation of ST/SC anchors on *S. oneidensis*

In this study, all genetic constructs were generated based on the plasmid pBAD202 encoding for the MtrF membrane protein of *S. oneidensis* as backbone.[40] The method of isothermal recombination, pioneered by Gibson in 2011,[53] was employed for the incorporation of large gene segments exceeding 100 bp. In order to enable sufficient recombination, all gene inserts possessed a sequence overlap of 30 to 50 base pairs with the vector backbone. To introduce short gene inserts, specific primers were used to directly integrate the desired characteristics into the vector backbone via PCR. A list of all primers used in this study can be found in table S5. The gene assembly was executed at 50 °C and 600 rpm for 1 hour. To remove any remaining unaltered vector backbone, DpnI digestion was conducted by adding 2 µL of DpnI (20 U/µL, New England Biolabs) into the solution. After 1 h incubation at 37 °C

and 600 rpm, 4 µL of the solution were used to transform *E. coli* Dh5α. Selection was accomplished by distributing 200 µL of cell suspension on a LB agar plate containing 50 µg/mL kanamycin. Plasmids of selected clones were multiped using overnight cultures of *E. coli* Dh5α at 37 °C and 180 rpm in LB medium containing 50 µg/mL kanamycin. Plasmid preparations were done using the using the ZR plasmid miniprep classic kit from Zymo Research. Verification of the gene sequence was performed through commercial sequencing (LGC Genomics).

pBAD202: Empty pBAD vector was purchased from Thermo Fisher as component of TOPO Cloning Kit

pBAD202_MtrF-Aest2-Myc: Primers SG117 and 118 were used to amplify the Esterase II gene from the plasmid pET-22b+_Aesterase2.[54] The primers had an overlap for the pBAD202_MtrF plasmid and a sequence segment to insert the Myc-tag to the C-terminus of the esterase during PCR. The plasmid pBAD202_MtrF was opened using the primers SG127 and SG128. The two DNA sequences were then assembled using isothermal recombination.

pBAD202_MtrF-SC-Myc: For introduction of the SC, PG37 and PG38 were utilized to excise the encoding sequence from a plasmid encoding for the Esterase II fused C-terminally to a SC-tag. A 30 bp overlap that was introduced at both termini of the gene enabled recombination of the SC in between the gene sequence of MtrF and Myc-tag of vector pBAD202_MtrF-Aest2-Myc that was linearized by primers PG39 and PG40 prior.

Transformation of *S. oneidensis*

For the transformation of different *S. oneidensis* strains, 25 mL of LB medium were inoculated with wild-type or nuclease deficient *S. oneidensis* from a cryostock stored at -80 °C and incubated overnight at 30 °C and 180 rpm. The next morning, this preculture was used to inoculate 25 mL of LB medium with a starting OD_{600} of 0.1. The cells were then cultivated to an OD_{600} = 0.8 and subsequently centrifuged at 10000xg. The cells were dissolved in an OD_{600} of approx. 2.5 and transferred to 1.5 mL reaction tubes. The cells were then washed 5 times with sterile-filtered sorbitol (1 M) at room temperature. 150 µL of cell suspension was then mixed with 200 ng of plasmid solution and transferred into an electroporation cuvette (PEQlab). Electroporation was performed at 1.2 kV and subsequently 200 µL of SOC medium (5 g/l yeast extract, 20 g/l tryptone, 10 mM $MgSO_4$, 10 mM $MgCl_2$, 2,5 mM KCl, 10 mM NaCl, 20 mM glucose) were added to the cells. After 1.5 h incubation at 30 °C and 600 rpm, 200 µL of the cell suspension was plated on an agar plate containing 50 µg/mL kanamycin and incubated overnight at 30 °C.

Verification of surface presentation of SC anchor proteins on *S. oneidensis* by immunofluorescence staining

To detect the presence of MtrF-SC-Myc fusion protein on the surface of *S. oneidensis*, cells were first freshly transformed with the corresponding plasmid. 25 mL of LB medium supplemented with kanamycin (final concentration 50 µg/mL) were inoculated by a clone and incubated for 8 h. 5 mL of the cell solution was aspirated by a syringe with an attached 0.8 mm cannula (Braun) and transferred into 100 mL of anoxic M4 medium (50 mM lactate (electron donor), 50 mM fumarate (electron acceptor) 1.27 mM K_2HPO_4, 0.73 mM KH_2PO_4, 5 mM HEPES, 2 mM $NaHCO_3$, 9 mM $(NH_4)_2SO_4$, 150 mM NaCl, 3.2 mM casamino acids, 0.1 mM

$CaCl_2$, 1 mM $MgSO_4$, 5 µM $CoCl_2$, 0.2 µM $CuSO_4$, 5.4 µM $FeCl_2$, 57 µM H_3BO_3, 1.3 µM $MnSO_4$, 67.6 µM Na_2EDTA, 3.9 µM Na_2MoO_4, 1.5 µM Na_2SO_4, 5 µM $NiCl_2$ and 1 µM $ZnSO_4$) supplemented with kanamycin in a final concentration of 50 µg/mL. The culture was incubated gas-tight overnight at 30 °C without shaking. The next morning, 100 mL of the anaerobic preculture were centrifuged at 4 °C and the cells were washed three times with ice-cold wash buffer (1.27 mM K_2HPO_4, 0.73 mM KH_2PO_4, 5 mM HEPES, 2 mM $NaHCO_3$, 9 mM $(NH4)_2SO_4$, 150 mM NaCl, 3.2 mM casamino acids, 0.1 mM $CaCl_2$, 1 mM $MgSO_4$, 5 µM $CoCl_2$, 0.2 µM $CuSO_4$, 5.4 µM $FeCl_2$, 57 µM H_3BO_3, 1.3 µM $MnSO_4$, 67.6 µM Na_2EDTA, 3.9 µM Na_2MoO_4, 1.5 µM Na_2SO_4, 5 µM $NiCl_2$ and 1 µM $ZnSO_4$). The cells were then adjusted to an OD_{600} of 3 and used to inoculate 100 mL of anoxic M4 medium including 50 µg/mL kanamycin to a starting OD_{600} of 0.15. The culture was then incubated gas-tight at 30 °C. After reaching an optical density of 0.5, protein expression was induced by adding arabinose to a final concentration of 10 mM. The cells were then incubated overnight at 30 °C without shaking. The cells were then harvested and washed with 1x PBS and adjusted to an OD_{600} of 5. Subsequently, 2 µL of anti-Myc antibody (DyLight488, Thermo Fisher) was added to 100 µL of cells and incubated for 45 min at room temperature at 600 rpm in the absence of light.[35, 36] The cells were then washed twice in 1x PBS and the fluorescence was measured with the Synergy MX plate reader at a wavelength of $490_{ex}/520_{em}$ nm. Microscopic imaging was performed using Zeiss Axiovert 200M at a magnification of 40x using FITC filter.

Verification of coupling efficiency of phi29-ST to SC anchor proteins on *S. oneidensis*

For evaluating the coupling efficiency of phi29-ST to *S. oneidensis*, cells were prepared as described above for immunofluorescence staining. Cells were then washed thrice with 1x phi29 reaction buffer and subsequently a final concentration of 10 µM of phi29-ST was added to 100 µL of a cell solution with an optical density of 5. Cells were then incubated for 1.5 h on ice and washed afterwards with 1x PBS three times. Fluorescent streptavidin conjugated to the dye AlexaFLuor488 was added in a final concentration of 0.15 mg/mL. After washing the cells again three times with 1x PBS, fluorescence was measured using Synergy MX plate reader and wavelengths of $490_{ex}/520_{em}$ nm.

Cell survival assay

To evaluate whether *S. oneidensis* can survive extended times in RCA reaction conditions, *S. oneidensis* SC and ΔN SC strains were prepared to present the SC surface anchor as described above. Cells were then adjusted to an optical density between 1-1.5 and distributed into for different reaction vessels in volumes of 500 µL and each cell solution was then washed thrice with a different ratio of M4 medium/phi29 reaction buffer (0/100, 25/75, 50/50, 100/0 %). 100 µL of cell solution were then transferred into a sterile 96 well plate. After sealing the plate with a sterile gas-impermeable foil (VWR), growth was monitored by measuring the optical density at a wavelength of 600 nm with the Tecan Spark plate reader (Tecan) at 30 °C. After 24 h of incubation, 5 µL of each well were transferred into a new well containing 95 µL fresh M4 medium supplemented with 50 µg/mL kanamycin but no added phi29 reaction buffer. Re-growth of cells was monitored again measuring the optical density at a wavelength of 600 nm with the Tecan plate reader at 30 °C for 48 h.

Circularization of RCA-template

The RCA process involved using the circular RCA template C, which was created by enzymatically ligating single-stranded oligonucleotides (See Table S6) with the T4 ligase. First, DNA template and primer were mixed to a final concentration of 5 µM both in 1X TE buffer (20 mM Tris-HCl, 1 mM EDTA, pH 7.6) supplemented with 100 µM NaCl. The solution was incubated for 1.5 h at 25 °C to allow for hybridization of the primer and template. Ligation mixture was prepared subsequently by adding 78 µL ddH_2O, 20 µl of 10x T4 ligation buffer (New England Biolabs, 500 mM Tris-HCl, 100 mM $MgCl_2$, 100 mM dithiothreitol, and 10 mM ATP, pH 7.6) and 2 µL of T4 ligase (400 000 U/mL, New England Biolabs). After incubation for 3 h at 25 °C and 400 rpm, ligation was stopped by heat denaturation for 20 min at 70 °C. After cooling to room temperature, samples were treated with exonuclease I (New England Biolabs, 40 U/µl) and III (New England Biolabs, 200 U/µL) to remove remaining linear DNA template. Digestion was performed by adding 24 µL of 10x NEB buffer 1 (New England Biolabs, 100 mM Bis-Tris-Propane-HCl, 100 mM $MgCl_2$, 10 mM dithiothreitol, pH 7.0) and 4 µL of each exonuclease to the sample. After incubation at 37 °C overnight and 600 rpm, digestion was stopped by heat denaturation at 80 °C for 20 min. Samples were washed five times with 0.1x TE buffer using vivacon ultracentrifugal filtration units (10 kDa cut-off, Sartorius). The DNA concentration of the sample was measured at 260 nm and concentration adjusted to 1 µM with 0.1x TE buffer. Purity of the template was evaluated by denaturing urea page electrophoresis. The template was then stored at -80 °C until further usage.

RCA standard conditions

To perform rolling circle amplification, RCA reaction mixtures were prepared by mixing circularized RCA template C, RCA primer and dNTPs (Jena Bioscience) at final concentrations of 360 nM, 300 nM and 2 mM respectively in 1x phi29 reaction buffer (New England Biolabs, 50 mM Tris-HCl,10 mM $MgCl_2$, 10 mM $(NH4)_2SO_4$ and 4 mM DTT, pH 7.5) supplemented with 0.1 mg/mL BSA. The RCA process was started by adding purified phi29-DNAP (standard RCA) in a final concentration of 20 nM or *S. oneidensis* cells (*in situ* RCA) functionalized with polymerase as described below and incubated at 30°C. Please note that other reaction conditions for RCA production are possible, especially when utilizing alternative polymerases,[56, 57] if required by the specific microorganism to be grown in the DNA hydrogel.

SYBR Green I (SG) based RCA-assay

To perform a fluorometric RCA-assay; SG I was added to RCA samples in a final concentration of 5x (stock solution 10000x concentrated, Thermo Fisher). Samples were then distributed in 25 µL aliquots into a 384 well plate and fluorescence measured with Synergy H1 plate reader (BioTek) at wavelengths of 494_{ex} and 521_{em} nm at 30°C for up to 72 h.

qPCR quantification of RCA product concentration

To quantify the concentration of the RCA product, we followed the protocol by Schneider *et al.*[42] RCA samples were collected at various time points (5 and 24 h) and the reaction was stopped by adding 90 µL of ddH_2O and heat denaturation at 80°C for 20 minutes. The samples were mixed thoroughly and subjected to ultrasonic treatment (VWR Ultrasonic Cleaner, 30 W) for 10 seconds to fragment the high molecular weight RCA product. Samples were then stored at -20°C and sonicated again before qPCR preparation. To determine the DNA concentration, a calibration curve of the amplicon sequence was created, ranging from 20 nM to 312.5 pM. RCA samples were diluted in ddH_2O (1:500 to 1:2000) based on reaction time and briefly sonicated again. 1.5 µL of a calibration or RCA sample were added to 20 µL of qPCR mix, prepared in 10 mL volumes with the following components: 1 mL 10x qPCR buffer(160 mM $(NH_4)_2SO_4$, 670 mM Tris-HCl, 25 mM KCl, 25 mM $MgCl_2$, 0.1% Tween-20)), 200 µL dNTPs (10 mM each), 100 µL each forward and reverse primer (100 µM), 20 µL TaqMan probe (100 µM), and 100 µL Taq DNA polymerase (5 U/µL), all mixed in autoclaved ddH_2O. qPCR was performed in triplicates on a Rotor-Gene RC6000 machine, starting with an initial denaturation at 95°C for 5 minutes, followed by 50 cycles of 30 seconds at 50°C for primer annealing, 20 seconds at 68°C for template extension, and 20 seconds at 95°C for denaturation. Fluorescence was monitored at wavelengths $470_{ex}/520_{em}$ nm. For quantification, ΔCt values were plotted against the logarithmic DNA concentration of the calibration samples. All sequences used are listed in Table S6.

Rheology measurements

To determine the apparent viscosity of DNA hydrogels, we employed the micromechanical indentation platform recently developed in our group, as described by Lemke *et al.*[43] This setup features a plunger measuring 18 mm in length and 4.3 mm in diameter, paired with a sample holder that has a cavity diameter of 4.5 mm and a depth of 4 mm. In a typical experiment, 75 µL of DNA hydrogel were placed into the sample holder, and the plunger was driven into the hydrogel at a rate of 1 mm/s, reaching a maximum penetration depth of 3.5 mm.

Stability assay of DNA-Hydrogels in the presence of wild-type *S. oneidensis* and nuclease deficient *S. oneidensis* strains

To evaluate the stability of pure DNA hydrogels in the presence of *S. oneidensis,* DNA hydrogels were prepared according to the conditions stated above. To allow microscopic visualization of the DNA Hydrogel, Cy5-dUTP was supplemented to the reaction mixture in a final dilution of 1:2000. 50 µL of the reaction mixture were transferred into a sterile flat bottom 96 (CellStar, Greiner-bio one) well plate or into 1.5 mL reaction vessels. After incubation for 72 h at 30 °C, DNA hydrogels were washed thrice with ddH_2O and five times with M4 medium containing 50 µg/mL kanamycin. *S. oneidensis* strains SC and ΔN SC that were grown anaerobically overnight in M4 medium containing 50 µg/mL kanamycin were used to inoculate DNA hydrogels in an OD_{600} of 0.5 by washing the hydrogels five times with cell solution. Images were then taken using LSM880 at a magnification of 10x. Argon laser channel HeNe3 (λ = 633 nm) was used to image the DNA hydrogel. After 24 h of incubation at 30 °C, a final concentration of 2.5 µM of 5-carboxyfluorescein diacetate (CFDA, Merck) was added to the cells and incubated for 1 h. Samples were then analyzed by microscopy to

illustrate the DNA hydrogel degradation and to depict the cells stained through the conversion of CFDA to carboxyl fluorescein. For DNA hydrogels produced in 1.5 mL reaction tubes, samples were taken to visualize the DNA amount with agarose gel electrophoresis after 24 h of incubation at 30 °C.

In situ rolling circle amplification with phi29-ST immobilized on *S. oneidensis* MtrF-SC

S. oneidensis SC and ΔN SC strains were cultivated overnight under anaerobic conditions in M4 medium containing 50 µg/mL kanamycin. The next morning cells were harvested by centrifugation at 10000xg at 4°C and then resuspended with an OD_{600} of 5. After washing the cells three times with 1x phi29 reaction buffer, phi29-ST was added to the cells at a final concentration of 10 µM. After incubation on ice for 1.5 hours, the cells were washed ten times with 1x phi29 reaction buffer. The cell suspension was then adjusted to an optical density of 2.5 and transferred to the RCA standard reaction mixture at a final dilution of 1:10. The cell-free supernatant from the final wash step as well as cells were no polymerase was added were used as a negative control. Unbound phi29-ST without cells served as a positive control. Samples were taken after 0, 5 and 24 hours of incubation and polymerisation was stopped by adding ethylenediaminetetraacetic acid (EDTA) at a final concentration of 100 mM. Furthermore, samples were centrifuged to remove cells and supernatant was taken for further analysis with agarose gel electrophoresis (1% w/v). For samples analysed with qPCR or micromechanical indentation no EDTA was added and samples analysed immediately with the respective methods.

Production of Ag/AgCl reference electrodes

Ag/AgCl reference electrodes for microfluidic BES chips were produced by electrochemical deposition of silver chloride on a silver wire. To achieve this, 3 cm of silver wire (Chempur, purity 99.99%) with a diameter of 0.8 mm connected to the working and working sense electrode port of the potentiostat 1010E (Gamry) was placed inside a platin mesh (0.06 mm wire diameter, 99.99 % purity, Chempur) that served as counter electrode. A commercial Ag/AgCl reference electrode SE10 (Sensortechnik Meinsberg) was placed in close proximity to the platin mesh and connected to the reference port of the potentiostat. After the setup was assembled, it was placed inside a 100 ml beaker and then filled up with degassed 0.1 M HCl. Electrochemical deposition of silver chloride was started by applying a potential of 0.05 V vs. EOC for 3000 s.

Production of PDMS chips for the microfluidic bioelectrochemical reactor

For the casting of a single microfluidic BES electrode chip or bubble trap 10 g of Sylgard 184 polydimethylsiloxane (PDMS, Sylgard) were mixed with 1 mL curing agent thoroughly in a falcon tube. After degassing the solution for 30 min in a desiccator, it was poured cautiously into the respective mould and placed into the oven for 2 h at 65-70 °C. Cured PDMS chips were then removed from the mold and subsequently plasma activated for surface bonding

using PlasmaFlecto machine (for 30 s at 0.02 bar and 100 % O_2, 300W). BES electrode chips were then bonded with plasma activated 1 mm thick microscope slides (VWR) whereas bubble traps were bonded with 1.5 mm thick stretches of plasma activated cured PDMS. Electrode chips were then equipped with a graphite electrode with a defined area of 0.3 cm^2. To enable electric connection with the potentiostat, copper foil was placed on the back of the graphite electrode and both coated with conductive silver lacquer. After the conductive silver lacquer had dried, 1 mL of twinsil silicone (picodent) was prepared by mixing solution A and B in a ratio of 1:1. The silicone was then very carefully spread over the back of the electrode and the copper wire to fix them permanently. After the silicone had hardened, freshly prepared 1g of PDMS including curing agent was poured over the electrode and the chip then incubated overnight at 65-70 °C in the oven.

Preparation of the microfluidic bioelectrochemical system

BES experiments in this study were performed using a polymethyl methacrylate (PMMA) frame (Figure S10) developed by the Gescher group that allows for the housing of three individual BES systems consisting of a bubble trap, an anode chip and a cathode chip.[44] The bubble trap and the anode chip were located in a sealable compartment that enabled the system to be gassed with nitrogen via El-flow select mass flow controller (Bronkhorst). Anode chips were further equipped with a 0.8 mm Ag/AgCl reference electrode and a 0.8 mm cannula (Braun) that allowed inoculation of the anode chip with bacterial cells or DNA hydrogel precursor material over 1.0 mm silicone tubes (Roth) from the side ports. The cathode chip was located in an oxic compartment to avoid the formation of hydrogen. Chips were equipped with 1.2 mm cannula and luer-lock adapter which allowed connection of the chips to the medium and waste reservoirs exterior to the frame via 1.0 mm silicone or PTFE tubing. To prevent the migration of bacteria into the medium reservoir during experiments, a 0.22 µm exchangeable sterile filter (VWR) was installed at the inlet position of the PMMA frame. The setup was then assembled via Fluigent Flow Unit L to a reservoir containing 70 % (v/v) ethanol. After connecting the reservoir with a 4 mm pneumatic tube to the Fluigent Flow control unit, the flow was started and the system flushed for 2 h at a flow rate of 100 µL/min. In the meantime, BES chips were connected to the 1010E potentiostat and the system closed by fastening the lid to the frame. Nitrogen flow was then started at a rate of 30 normliter/min and the reservoir changed from ethanol to M4 chip medium (20 mM lactate (electron donor), 1.27 mM K_2HPO_4, 0.73 mM KH_2PO_4, 5 mM HEPES, 2 mM $NaHCO_3$, 9 mM $(NH4)_2SO_4$, 150 mM NaCl, 3.2 mM casamino acids, 0.1 mM $CaCl_2$, 1 mM $MgSO_4$, 5 µM $CoCl_2$, 0.2 µM $CuSO_4$, 5.4 µM $FeCl_2$, 57 µM H_3BO_3, 1.3 µM $MnSO_4$, 67.6 µM Na_2EDTA, 3.9 µM Na_2MoO_4, 1.5 µM Na_2SO_4, 5 µM $NiCl_2$ and 1 µM $ZnSO_4$) supplemented with 50 µg/mL kanamycin.

Cultivation of *S. oneidensis* in the microfluidic BES

After assembly, the system was equilibrated over night to remove remaining oxygen. Chronoamperometric measurements were started by applying a potential of -199 mV vs. Ag/AgCl. Once current reached stable values close to 0 A, the designated *S. oneidensis* strain for the experiment was prepared for inoculation of the BES. For this, cells grown over night in 100 mL anoxic M4 medium containing 50 µg/mL kanamycin were harvested by centrifugation at 4000xg for 30 min and 4 °C. Cells were washed thrice with washing buffer

and the cell solution adjusted to an optical density of 2.0 after the last wash step. Cell solutions were then inoculated into the system using KR analytics LTP Nexus 3000 syringe pump at a flow rate of 2 ml/h for 2 h while setting the medium flow rate of the BES to 2 mL/h. Cells were then grown at 30 °C at a medium flow rate of 4 mL/h for at least 5 d or until the current values reached a stable plateau. Experiments with the aim to create DNA hydrogels were continued by adding arabinose in a final concentration of 10 mM to the chip medium. After 24 h medium flow was stopped and the system was flushed for 30 min with 1x phi29 reaction buffer. 1000 µL RCA mixture was then injected together with 20 nM of phi29-ST into the system at a flow rate of 2 mL/h. After injecting all of the polymerisation solution into the BES, the system was incubated for 24 h at 30 °C. M4 chip medium without arabinose was then flushed again through the system at a flow rate of 4 mL/h. Experiments were terminated after another 5 days of cultivation and the BES chips disintegrated from the system. To stain DNA potentially generated on the surface of electrodes, chips were then flushed with 1 mL 0.5x SYBR Green I solution followed by 1 mL 1x PBS. Chips were then investigated using LSM880 at 10x magnification and argon laser (λ = 488 nm) and DPSS 561-10 laser (λ = 560 nm) for monitoring the graphite electrode surface.

Cultivation of *S. oneidensis* in parallelised standard-BES

Investigations of current production and hydrogel formation in Standard-BES was performed using an eightfold e-Cuvette system previously developed.[45, 46] Each Cuvette can harbour a screen-printed gold electrode (C220 AT, Deutsche Metrohm GmbH & Co. KG, Germany) including anode and cathode. The screen-printed electrodes were inserted into an 3D-printed lid made from Rigur RGD450 resin in an SLA printer (Stratasys, USA) hosting peripheral attachments for cable and gas line connection. Afterwards, the lid was placed on eight cuvettes filled with M4 BES-standard medium (50 mM lactate (electron donor), 1.27 mM K_2HPO_4, 0.73 mM KH_2PO_4, 5 mM HEPES, 2 mM $NaHCO_3$, 9 mM $(NH_4)_2SO_4$, 150 mM NaCl, 3.2 mM casamino acids, 0.1 mM $CaCl_2$, 1 mM $MgSO_4$, 5 µM $CoCl_2$, 0.2 µM $CuSO_4$, 5.4 µM $FeCl_2$, 57 µM H_3BO_3, 1.3 µM $MnSO_4$, 67.6 µM Na_2EDTA, 3.9 µM Na_2MoO_4, 1.5 µM Na_2SO_4, 5 µM $NiCl_2$ and 1 µM ZnSO4) supplemented with 50 µg/mL kanamycin. Ag/AgCl reference electrodes (Pine Research Instrumentation Inc., US) were inserted via a port in the lid completing the three-electrode setup. Gas connection was established via sterile cannulas (Ø 0.6 mm, B. Braun, Germany) inserted through the lid allowing to sparge the e-cuvettes with nitrogen in a flow rate of 5 normliter/min. After 30 min, connection to the 8-channel potentiostat (Palmsens BV, Netherlands) was established and measurement started by setting the potential at -199 mV vs. Ag/AgCl reference electrode. After 2 h of equilibration, four e-cuvettes were inoculated by either *S. oneidensis* SC or ΔN SC with a final OD_{600} of 0.5. After 24 h of incubation, fresh medium supplemented with 10 mM arabinose for induction was added to the cells. The next morning, medium was removed in all e-cuvette. Subsequently, two e-cuvette of each strain used were incubated with phi29-ST in a final concentration of 10 µM in 1x phi29 reaction buffer. Two e-cuvette BES of each strain served as negative control and were filled up with only 1x phi29 reaction. After 30 min of incubation, all e-cuvettes were washed thrice with 1x phi29 reaction buffer and afterwards filled with RCA reaction mixture containing 2 mM of dNTP mixture, 300 nM Template C, 360 nM Primer C, 0.1 mg/mL BSA as well as 1x phi29 reaction buffer. After another 24 h of incubation, RCA mixture was exchanged with fresh M4 BES-standard medium and incubated over-night. The setup was then disassembled and the electrodes stained for biofilm visualization with 0.5x SYBR Safe

in PBS. Electrode surface was then investigated with LSM 880 at 10x magnification using argon laser (λ = 488 nm) for monitoring of SYBR Green I and DPSS 561-10 laser (λ = 560 nm) for monitoring the gold electrode surface.

Supporting Figures

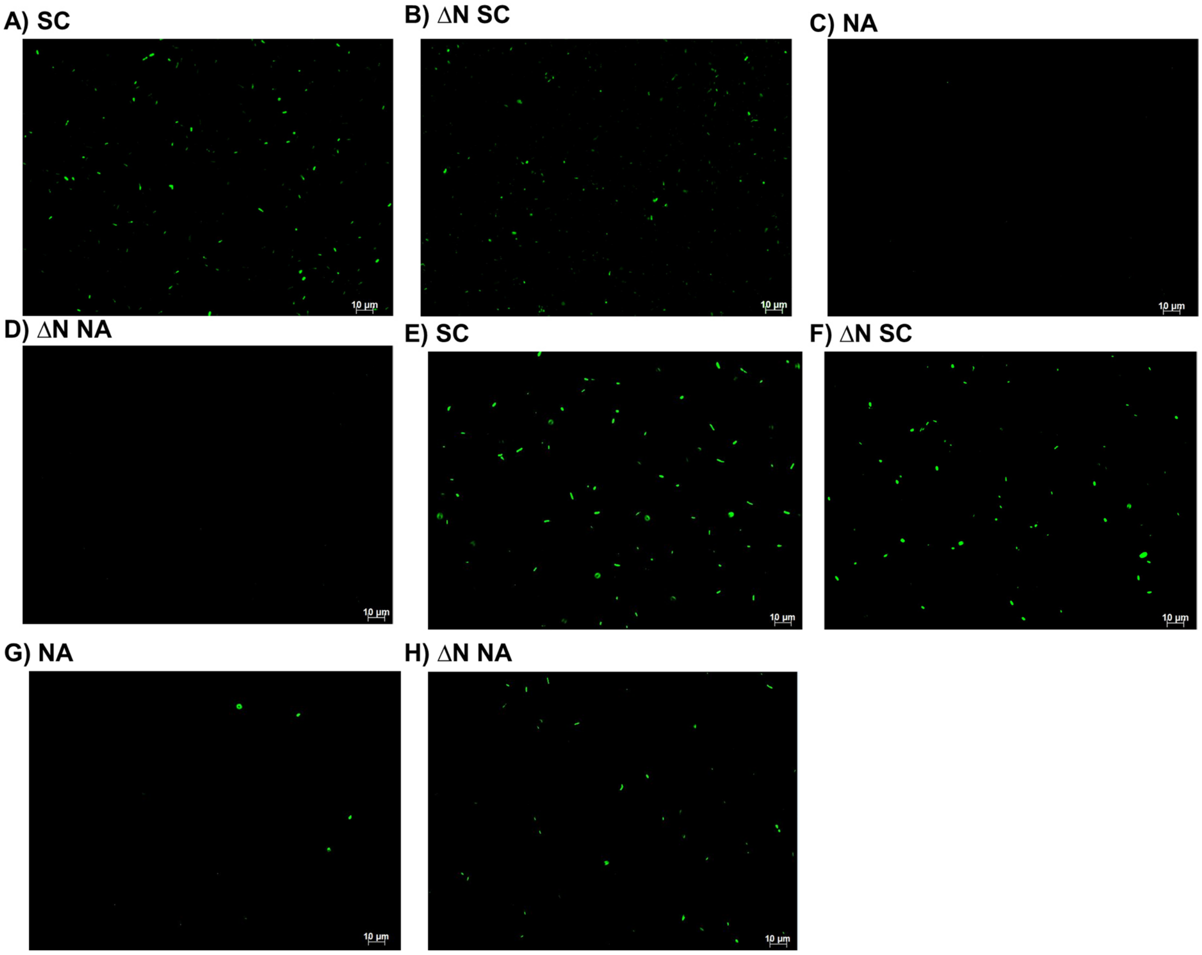

Figure S1: Evaluation of SC surface presentation and coupling functionality of phi29-ST via microscopic imaging. Stains harbouring only the empty pBAD vector (NA and ΔN NA) served as control. A-D) For evaluation of SC surface presentation cells prepared as decribed in the Materials and Methods section were incubated with fluorescent anti-Myc antibody$_{DL488}$ and investigated at 40x magnification using the FITC-Channel. Microscopic images clearly show that only strains harbouring the plasmid for MtrF-SC surface anchor are stained, thus indicating succesfull surface presentation. E-H) To evaluate coupling functionality of SC surface anchors, cells were incubated with phi29-ST polymerase. Subsequently, STV$_{AF488}$ was added which binds to the SBP-Tag of the phi29-ST polymerase. Cells were again investigated at 40x magnification using FITC channel. Note that for strains SC and ΔN SC plenty of fluorescent cells can be observed after staining. In contrast to the antibody staining described in A-D, both control strains also show some fluorescent cells. Since the amount appeared to be less compared to strains SC and ΔN SC these results suggested that specific coupling of phi29-ST to SC on cells but unspecific binding e.g. due to polymerase binding at DNA present also takes place.

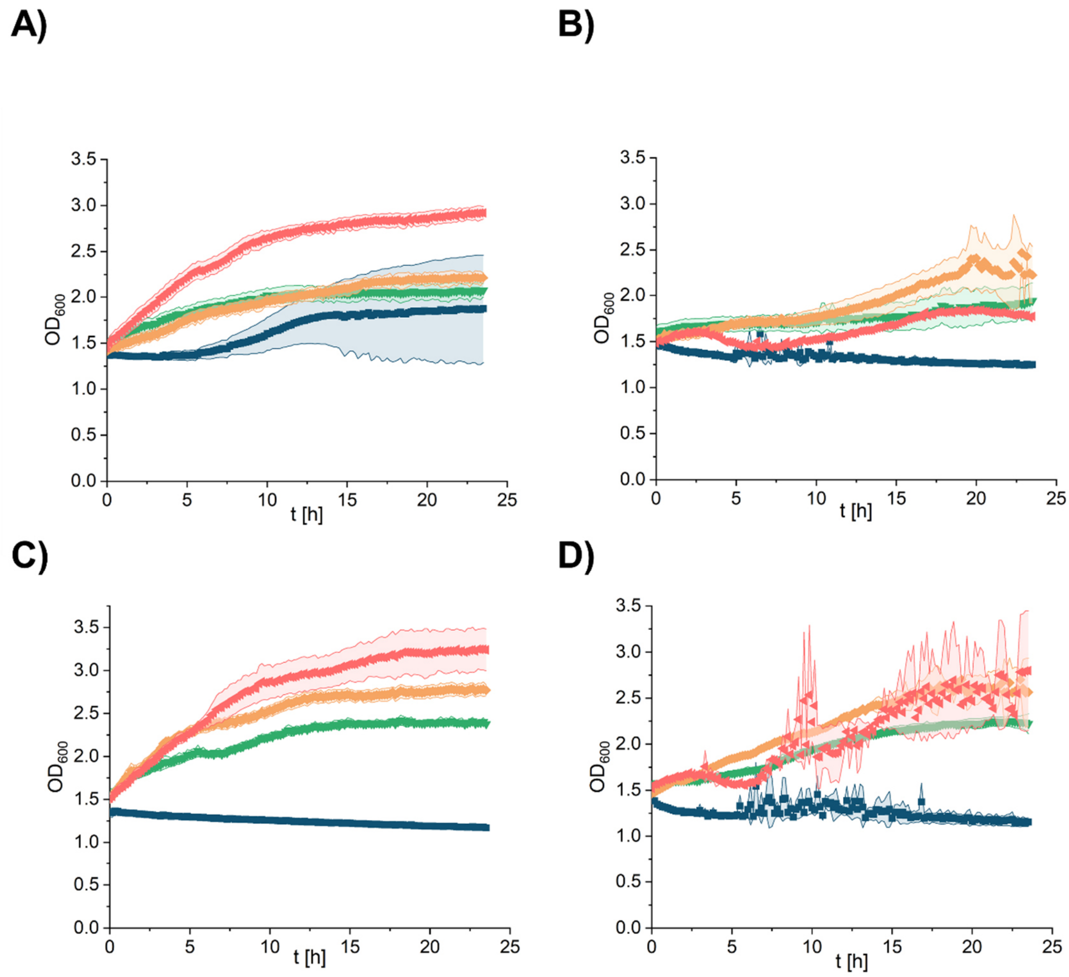

**M4 medium (%)**	0	25	50	100
**phi29 reaction buffer (%)**	100	75	50	0

Figure S2: Growth of *S. oneidensis* strains SC and ΔN SC in M4 medium containing different ratios phi29 reaction buffer. A, B) SC and ΔN SC incubated over 24 h in M4 medium supplemented with phi29 reaction buffer (containing DTT) in ratios of 0/100, 25/75, 50/50 and 100/0 %. C, D) SC and ΔN SC incubated over 24 h under the same conditions stated above but using phi29 reaction buffer (without DTT). Cells of both strains appeared to grow better in conditions without DTT indicating that this reducing molecule has an inhibiting influence on *S. oneidensis* growth. Overall, cells incubated in conditions with a higher share of M4 medium in the mixture grew faster and reached a higher optical density as expected. Cells incubated without any medium were not able to grow but OD_{600} remained relatively stable over a period of 24 h.

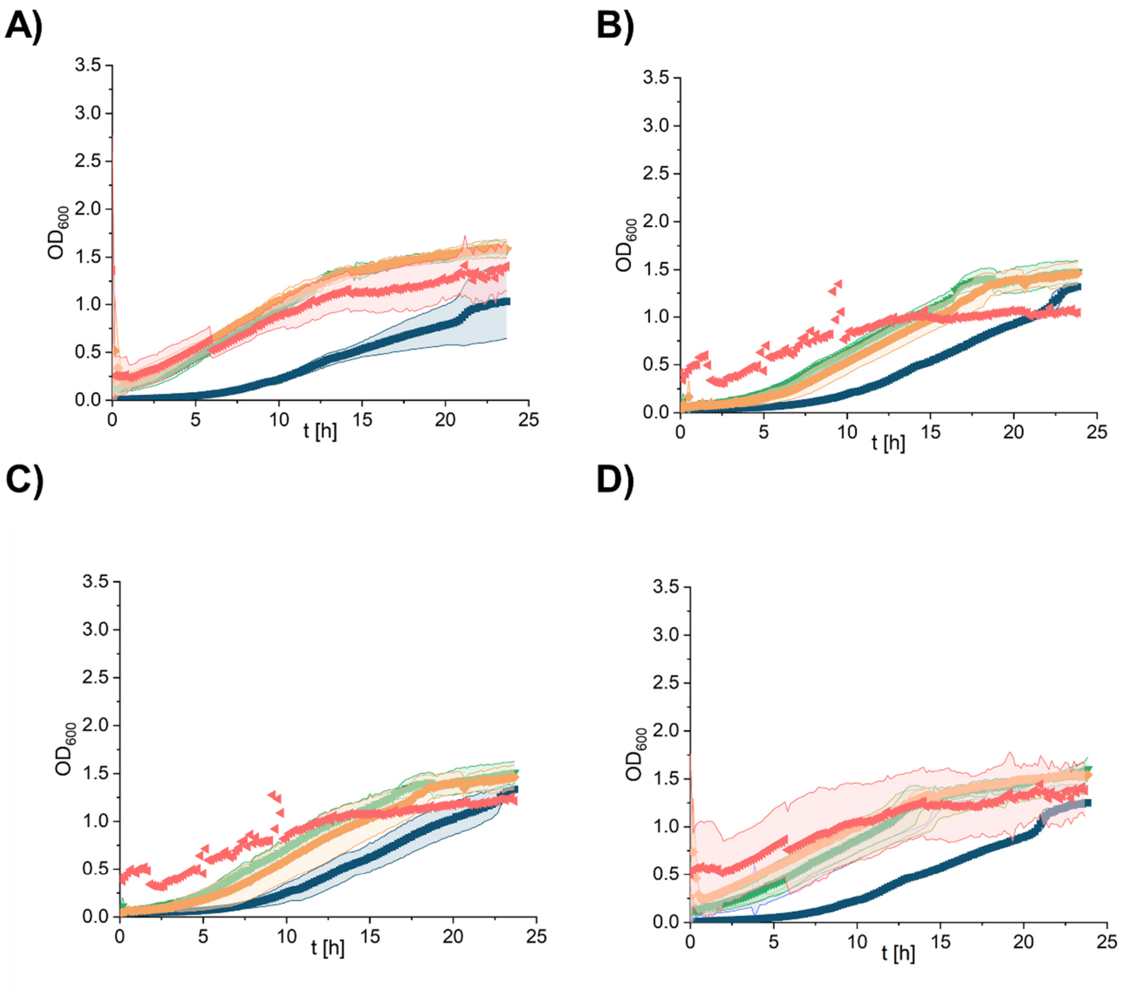

**M4 medium (%)**	0	25	50	100
**phi29 reaction buffer (%)**	100	75	50	0

Figure S3: Regrowth of *S. oneidensis* in 100% M4 medium after 24 h incubation under the different conditions investigated in Figure S2. After 24 h of incubation, 5 µL of cells from each tested condition were transferred into 100 µL of fresh M4 medium lacking any phi29 reaction buffer, and OD_{600} was monitored for 72 h. Strains SC and ΔN SC that were incubated in different ratios of M4 medium and phi29 reaction buffer with DTT (A, B) or without DTT in the reaction buffer (C, D). Note that cells incubated in all tested conditions were able to regrow after a few hours. Also note that the observed lag time was highest for cells that were incubated previously in pure 100 % phi29 reaction buffer.

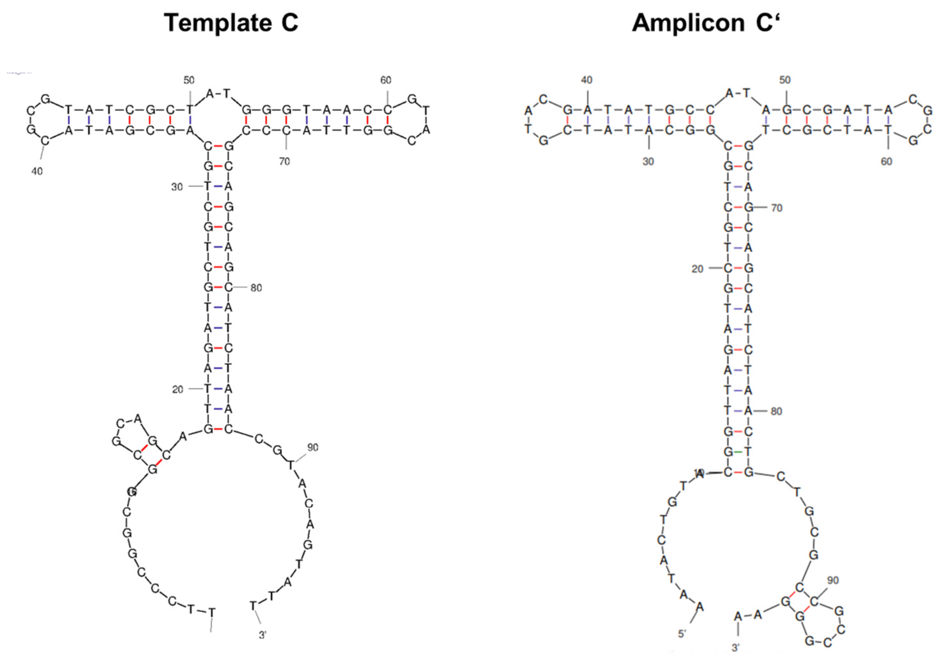

**Free Energy [∆G]**	-30,7 kcal/mol	-31,57 kcal/mol
**Intramolecular base pairing**	65,3 %	69,4 %

Figure S4: Secondary structure prediction of the circular DNA template C and its complementary amplicon product C'**.** For calculations mfold software was used to find the respective structure with the lowest free energy.[58]

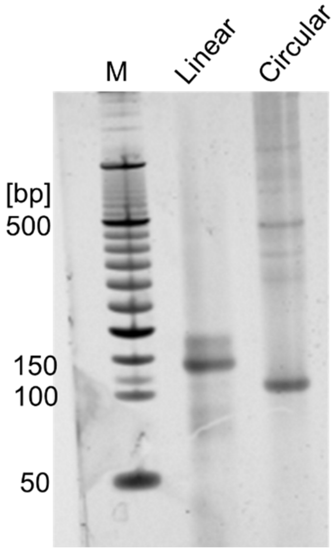

Figure S5: Characterization of circular template C after ligation and purification. 2 µM of circularized template purified template C was loaded onto 12% Urea-PAGE. 2 µM of linear precursor DNA served as control. Note that for the lane where circular template C was loaded onto the gel a prominent band at approximately 100 bp can be observed. The higher electrophoretic mobility of this band than that of the linear oligomer indicates that the ligation was successful. Note that additional bands of higher molecular weight are most likely ligated dimer and trimer structures. M: 50 bp Orange Ruler DNA ladder.

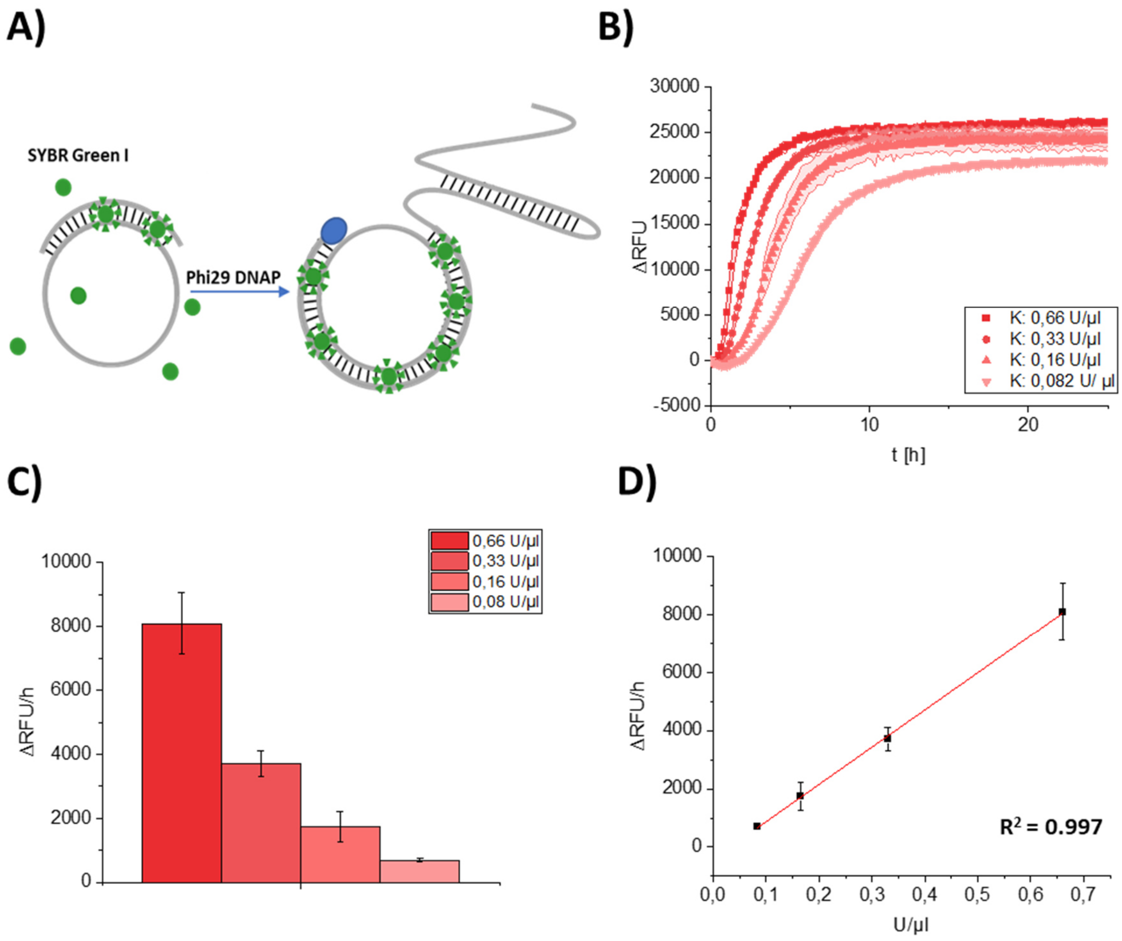

Figure S6: Activity screen of different commercial phi29 concentrations with a fluorescence-based rolling circle activity assay. A) Principle of the fluorescence-based RCA assay. A primed circular DNA template serves as a starting point for RCA mediated by the phi29-DNAP. Once the polymerase reaches the primer again, it separates the previously synthesized DNA from the template strand. The resulting long ssDNA strand folds into dsDNA due to its sequence-specific Watson-Crick base-pairing properties. This leads to the incorporation of SYBR Green I (SG), which results in a strong increase in fluorescence that can be measured by a florescence plate reader. RCA mixture prepared under conditions stated in the Materials and Methods section was added with varying amounts of commercial phi29-NEB and fluorescence measured at λ ($494_{ex}/521_{em}$). B) Course of fluorescence over a time period of 25 h for different amounts of phi29-NEB. C) Initial increase in fluorescence intensity (slope of curves in B) obtained for various commercial phi29 DNA polymerase concentrations. D) Linear regression of SG fluorescence slope and amount of used phi29-DNAP (R^2= 0,997). Note that the results indicate that the assay is well suited to distinguish different activities of phi29 DNAP.

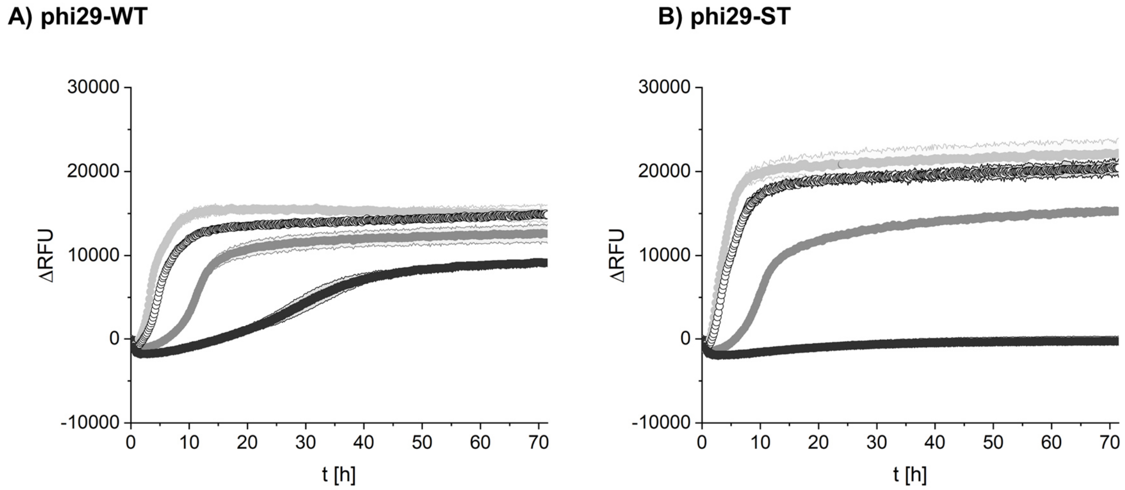

DTT [4 mM]	+	+	-	-
M4 medium [25%]	-	+	-	+

Figure S7: Activity screen of different phi29-WT and phi29-ST variants under varying reaction conditions (A and B). RCA reaction conditions varied by using reaction buffer with 4 mM DTT (standard conditions) or reaction buffer without DTT. Additionally, either no or 25 % M4 medium was supplemented to the RCA reaction mixtures. The results show that RCA performed under standard conditions leads the strongest increase in fluorescence for both phi29-DNAP variants, whereas samples containing reaction buffer without DTT show a slightly reduced activity. Note that adding M4 medium seems to impair the RCA process significantly with almost complete suppression for the condition where also no DTT was present. The observed results might be due to an impaired binding of the polymerase to the DNA since M4 medium contains 150 mM NaCl which weakens DNA binding. Likewise, the binding of SG I dye might be impaired due to the presence of higher salt concentrations which would result in lower fluorescence recorded. Given these potential artefacts, we decided to analyze the influence of variable conditions with qPCR and rheology (Figure 3, main text) as these methods are independent on an intercalating dye.

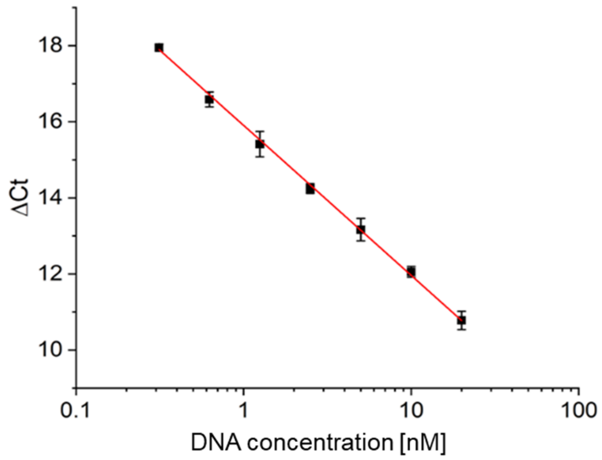

Figure S8: Representative calibration curve obtained by qPCR to quantify the concentration of RCA products. Linear regression analysis was conducted with the Ct values obtained from various concentrations of linear single-stranded DNA, which corresponded to a single amplicon of the circular template used in RCA.

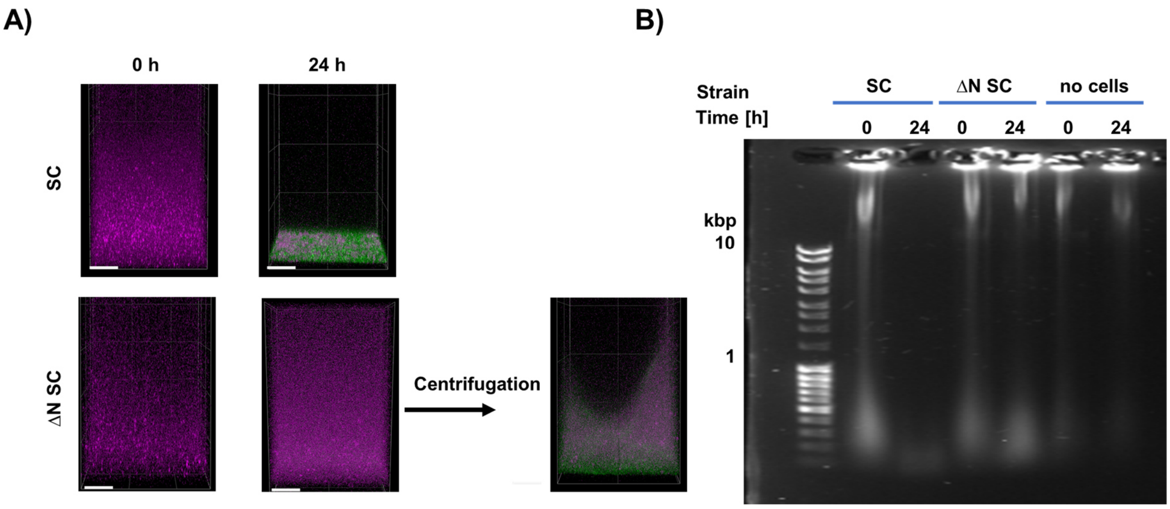

Figure S9: Stability of pure DNA hydrogel in the presence of the strains SC and nuclease deficient ΔN SC. A) An anaerobic culture of both *S. oneidensis* strains was grown over night, and subsequently inoculated into a well containing the Cy5 stained DNA hydrogel. Microscopy investigations of the DNA hydrogels were carried out to investigate the morphology of the DNA hydrogel material (purple). After 24 h of incubation 5-Carboxyfluorescein diacetate (CFDA) which is converted to 5-(6)carboxyfluorescein by metabolically active bacteria was added and samples again analysed using argon laser for illustrating cells (green) and HeNE laser for depicting the DNA hydrogel (purple).Since only a thin layer of cell and material could be observed at the bottom of the well, the results indicated that wild-type *S. oneidensis* almost completely breaks down the DNA hydrogel material. In contrast for wells incubated with strain ΔN SC, a prominent hydrogel up to 1 mm in height but no cells could be observed initially. Samples were therefore centrifuged and investigated again. Here, compressed material and cells were clearly visible, indicating that the strain ΔN SC promotes hydrogel stability. Scale bars represent 200 µm. B) Hydrogel samples incubated for 0 or 24 h with both *S. oneidensis* strains in 1.5 mL reaction tubes were loaded onto 1% agarose gel stained with SYBR Safe. Note that wild-type *S. oneidensis* degrades the DNA hydrogel completely whereas nuclease deficient strain promotes DNA hydrogel stability.

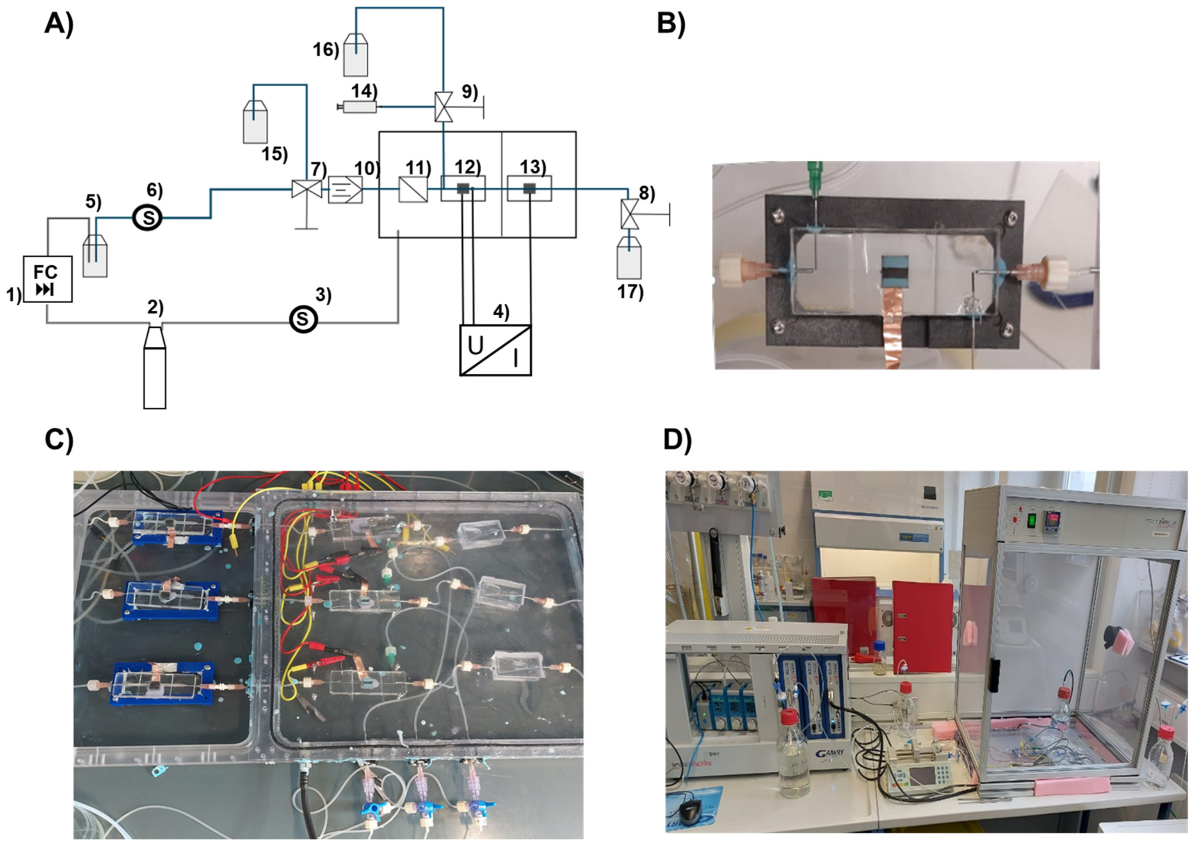

Figure S10: Overview of the microfluidic BES-system.[44] A) Flow chart of the BES-system with all components used. 1) Fluigent Flow Ez™ controller. 2) Nitrogen supply. 3) Bronkhorst EL-Flow Select gas mas flow controller. 4) Gamry potentiostat 1010E. 5) Pressure driven anaerobic medium reservoir. 6) Fluigent Flow unit. 7-9) Three-port-valve. 10) Sterile filter. 11) Bubble-trap. 12) Anode chip with integrated Ag/AgCl reference electrode. 13) Cathode chip. 14) Side port for inoculation of the system. 15-17) Waste. B) BES anode chip with integrated graphite electrode and Ag/AgCl reference electrode. The chip is connected to the microfluidic system via cannulas (orange) or inoculated with cells/DNA hydrogel precursor material over a different cannula (green). C) BES frame with three individual anode chips (anoxic compartment) and three cathode chips. The individual electrodes are connected to the potentiostat via copper leads. D) Complete setup with flow controller, potentiostat, BES frame, incubation chamber and syringe pump for the inoculum.

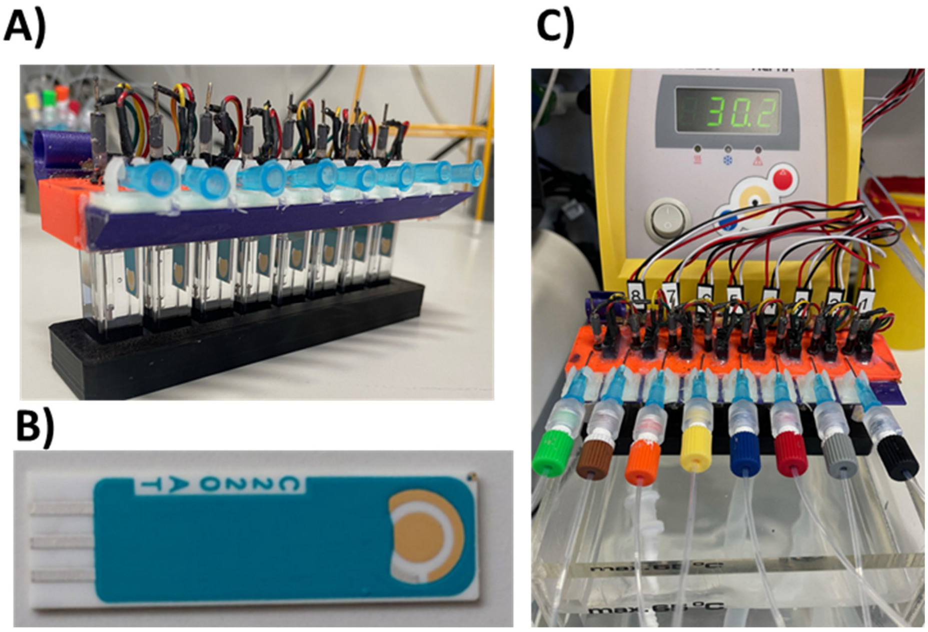

Figure S11: Overview over the e-cuvette system[45, 46] used for BES-batch experiments. A) The e-cuvette system can harbour up to eight screen-printed gold electrodes (B) in 4 mL cuvettes allowing parallelized measurements. C) After integration of screen-printed electrode into the system, connection to gas flow and potentiostat can be achieved using connection ports of a 3D-printed lid. The whole system is placed in a water bath allowing for temperature control.

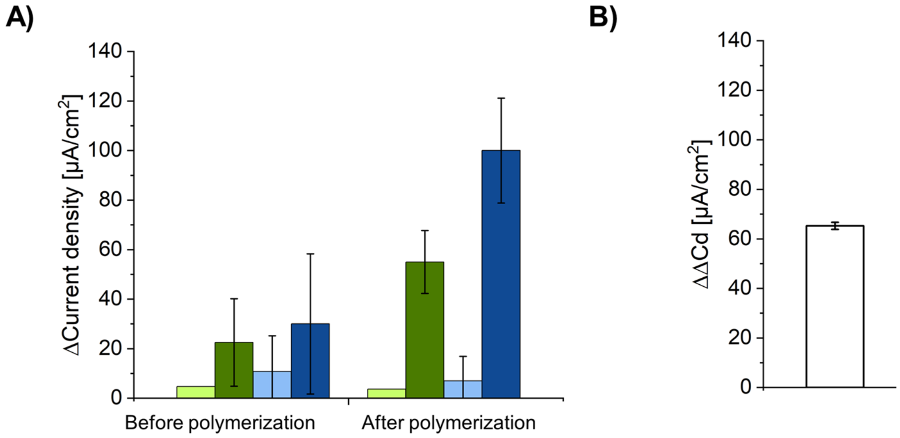

	S. oneidensis strain	Polymerase
	SC	-
	SC	+phi29-ST
	ΔN SC	-
	ΔN SC	+phi29-ST

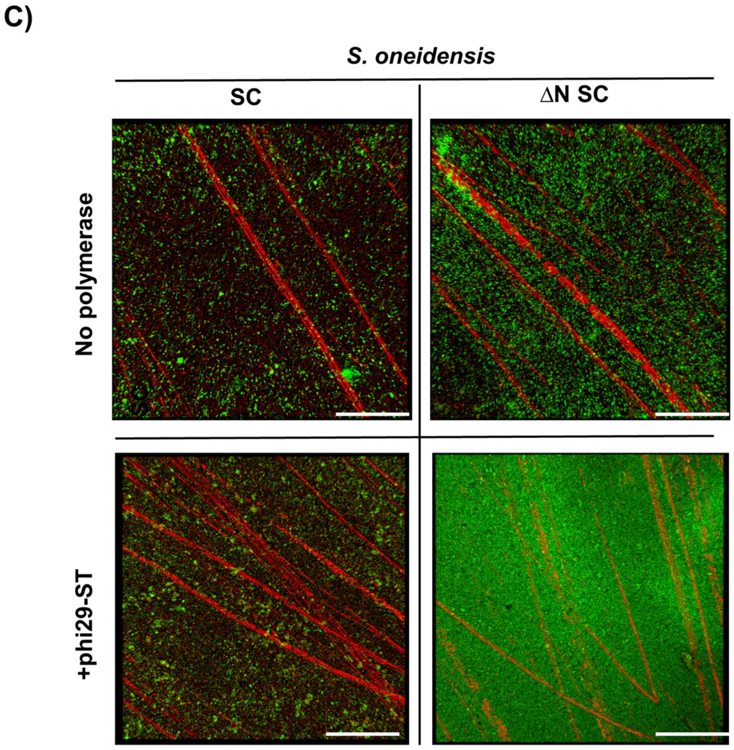

Figure S12: Assessment of current density and DNA hydrogel formation morphology using *S. oneidensis* strains SC and ΔN SC in a batch-operated E-cuvette BES equipped with eight parallelized, independent screen-printed gold electrodes (Figure S11). A) Strains SC and ΔN SC were each inoculated into four independent BES at a potential of -199 mV vs. Ag/AgCl. After 24 h of cultivation, cells were induced for presentation of SC surface anchor. Subsequently, M4 BES-standard medium was removed and electrodes were incubated with 10 µM phi29-ST for 30 min. BES without added polymerase served as negative control. Chamber were then washed, RCA reaction mixture was added and incubated for 24 h (for details, see Methods section above). The reaction mixture was then removed and all chambers were filled with fresh M4 BES-standard medium and incubated over-night. Maximum current for different conditions tested were extracted for both strains before and after polymerization and plotted as bar chart for comparison. Standard deviations are derived from

biological duplicates. The results show that strains SC and ΔN SC produce comparable amounts of current before polymerization. After polymerization, a steep increase can be observed for strain ΔN SC, reaching significantly higher current values of around 100 ± 20 µA/cm2 than strain SC. B) Difference in current density after polymerization for different independent BES inoculated with strain ΔN SC and incubated either with or without phi29-ST. The results clearly indicate that polymerization in the BES leads to increased current. C) Microscopic images of electrode surfaces (red) used in BES experiments shown in (A). Scale bars are 200 µm. Cells were strained with SYBR Green I for visualization (green). Note that the ΔN SC electrode surfaces in general show more dense biofilm formation than those with wild-type strain SC, presumably due to the accumulation of extracellular DNA, even without polymerization. However, electrode surface of the BES where strain ΔN SC was incubated with polymerase shows complete coverage of the electrode with a dense biofilm. Hence, the microscopic investigations align very well with current values shown in (A).

Supplementary Tables

Table S1: Sequences of primers used for the deletion of the nucleases ExeS, ExeM and EndA

Primer	Sequence 5' → 3'
3622_ExeS_500up_F	GTGCCAAGCTTGCATGCCTGCAGGTCGACAGCCA AGCAAAATACG
3623_ExeS_500up_R	GATATCAAAAGAAAGAGGCTAATGCGTGACCCCT AAAAAGTATCG
3624_ExeS_500Down_F	AAAACACGATACTTTTTAGGGGTCACGCATTAGCC TCTTTCTTTT
3625_ExeS_500Down_R	CGAATTCGAGCTCGGTACCCGGGGATCTCTCTAC TGTAGGGGATC
3626_ExeM_500Up_F	CCAAGCTTGCATGCCTGCAGGTCGAAATCCCACA GTTGTTTTAAT
3627_ExeM_500Up_R	CAACCTAGTCGTGAGAGTGCAAACAGTATCAACA ACCCCATCATT
3628_ExeM_500Down_F	GCAATATTATAATGATGGGGTTGTTGATACTGTTT GCACTCTCAC
3629_ExeM_500Down_R	AGGCAGTTTCGGTATTAGGTAGTGACGCGTCACT ACCTAATACCG
3634_EndA_500Down_F	TTGGAACTCTCATTGTCGACCTTATCCCAAAAATA ATCAGTACTA
3635_EndA_500Down_R	GATTACGAATTCGAGCTCGGTACCCGGGGATCGT GCAGGCACAGC
3636_EndA_500Up_R	GCGTAGTACTGATTATTTTTGGGATAAGGTCGACA ATGAGAGTTC
3637_EndA_500Up_F	CAGTGCCAAGCTTGCATGCCTGCAGGTCGAGGTC GGTTCGCTACA

Table S2: Genetic constructs used in this work

Plasmid	Genetic construct	Source
**pBAD_MtrF**	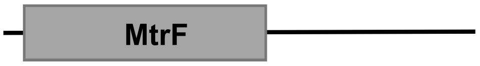	[40]
**pBAD_MtrF-Aest2-Myc**	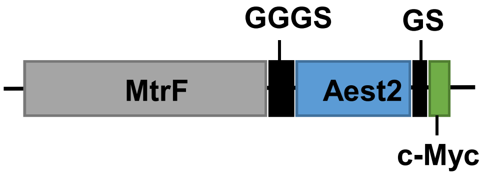	This study
**pBAD_MtrF-SC-Myc**	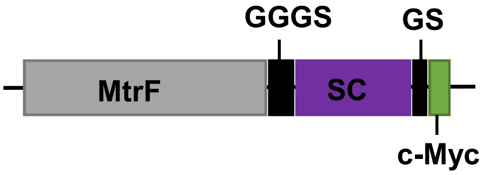	This study
**pET22b+_phi29-ST**	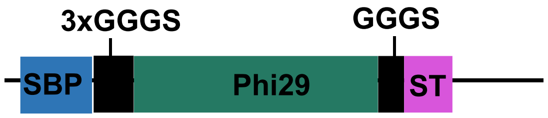	[41]

Table S3: Amino acid sequences of MtrF-fusion proteins used in this study

Functional protein	Amino acid sequence
**MtrF-Aest2-Myc**	MNKFASFTTQYSLMLLIATLLSACGGSDGDDGSPGEPGKPPAM<br>TISSLNISVDKVAISDGIAQVDYQVSNQENQAVVGIPSATFIAAQL<br>LPQGATGAGNSSEWQHFTSETCAASCPGTFVDHKNGHYSYRF<br>SATFNGMNGVTFLSDATQRLVIKIGGDALADGTVLPITNQHYDW<br>QSSGNMLAYTRNLVSIDTCNSCHSNLAFHGGRYNQVETCVTCH<br>NSKKVSNAADIFPQMIHSKHLTGFPQSISNCQTCHADNPDLADR<br>QNWYRVPTMEACGACHTQINFPAGQGHPAQTDNSNCVACHN<br>ADWTANVHSNAAQTSALAQFNASISSASMDANGTITVAVSLTNP<br>TTGTAYADSADKLKFISDLRIYANWGTSFDYSSRSARSIRLPEST<br>PIAGSNGTYSYNISGLTVPAGTESDRGGLAIQGRVCAKDSVLVD<br>CSTELAEVLVIKSSHSYFNMSALTTTGRREVISNAKCASCHGDQ<br>QLNIHGARNDLAGQCQLCHNPNMLADATATNPSMTSFDFKQLI<br>HGLHSSQFAGFEDLNYPGNIGNCAQCHINDSTGISTVALPLNAA<br>VQPLALNNGTFTSPIAAVCSNCHSSDATQNHMRQQGAVFAGTK<br>ADATAGTETCAFCHGQGTVADVLKVHPINGGGGSPLDPVIQQV<br>LDQLNRMPAPDYKHLSAQQFRSQQSLFPPVKKEPVAEVREFD<br>MDLPGRTLKVRMYRPEGVEPPYPALVYYHGGGWVVGDLETHD<br>PVCRVLAKDGRAVVFSVDYRLAPEHKFPAAVEDAYDALQWIAE<br>RAADFHLDPARIAVGGDSAGGNLAAVTSILAKERGGPAIAFQLLI<br>YPSTGYDPAHPPASIEENAEGYLLTGGMMLWFRDQYLNSLEEL<br>THPWFSPVLYPDLSGLPPAYIATAQYDPLRDVGKLYAEALNKAG<br>VKVEIENFEDLIHGFAQFYSLSPGATKALVRIAEKLRDALAGEQK<br>LISEEDL**
**MtrF-SC-Myc**	MNKFASFTTQYSLMLLIATLLSACGGSDGDDGSPGEPGKPPAM<br>TISSLNISVDKVAISDGIAQVDYQVSNQENQAVVGIPSATFIAAQL<br>LPQGATGAGNSSEWQHFTSETCAASCPGTFVDHKNGHYSYRF<br>SATFNGMNGVTFLSDATQRLVIKIGGDALADGTVLPITNQHYDW<br>QSSGNMLAYTRNLVSIDTCNSCHSNLAFHGGRYNQVETCVTCH<br>NSKKVSNAADIFPQMIHSKHLTGFPQSISNCQTCHADNPDLADR<br>QNWYRVPTMEACGACHTQINFPAGQGHPAQTDNSNCVACHN<br>ADWTANVHSNAAQTSALAQFNASISSASMDANGTITVAVSLTNP<br>TTGTAYADSADKLKFISDLRIYANWGTSFDYSSRSARSIRLPEST<br>PIAGSNGTYSYNISGLTVPAGTESDRGGLAIQGRVCAKDSVLVD<br>CSTELAEVLVIKSSHSYFNMSALTTTGRREVISNAKCASCHGDQ<br>QLNIHGARNDLAGQCQLCHNPNMLADATATNPSMTSFDFKQLI<br>HGLHSSQFAGFEDLNYPGNIGNCAQCHINDSTGISTVALPLNAA<br>VQPLALNNGTFTSPIAAVCSNCHSSDATQNHMRQQGAVFAGTK<br>ADATAGTETCAFCHGQGTVADVLKVHPINGGGGSVDTLSGLSS<br>EQGQSGDMTIEEDSATHIKFSKRDEDGKELAGATMELRDSSGK<br>TISTWISDGQVKDFYLYPGKYTFVETAAPDGYEVATAITFTVNEQ<br>GQVTVNGKATKGDAHIGEQKLISEEDL**

Table S4: Strains used in this study

**Strain name**	**Genomic genotype**	**Relevant genotype on pBAD plasmid**	**Source**
Wild-type	Wild-type		[12]
NA	Wild-type	Kan^R,P_{ara},	This study
SC	Wild-type	Kan^R,P_{ara}, MtrF-SC-Myc	This study
Nuclease deficient (ΔN)	Δ*ExeM,* Δ*ExeS,* Δ*EndA*		Generated according to [37,38]
ΔN NA	Δ*ExeM,* Δ*ExeS,* Δ*EndA*	Kan^R,P_{ara},	This study
ΔN SC	Δ*ExeM,* Δ*ExeS,* Δ*EndA*	Kan^R,P_{ara}, MtrF-SC-Myc	This study

Table S5: Sequences of primer used for this work

**Primer**	**Sequence 5' → 3'**
SG_119	TGAGCCACCTCCGCCGTTTATTGGATGGACTTTGAGTACGTCG
SG_120	GGTGAACAGAAACTGATCAGCGAAGAAGATCTGTAATAAACGGTCTCCAGCTTGGCTG
SG_127	ATCTTCTTCGCTGATCAGTTTCTGTTCACCGGCCAGCGCGTCTCGAAG
SG_128	GTCCATCCAATAAACGGCGGAGGTGGCTCACCGCTCGATCCCGTCATTCAG
PG37	GGTGAACAGAAACTGATCAGCGAA
PG38	GTTTATTGGATGGACTTTGAGTACGTCGGC
PG39	CGTACTCAAAGTCCATCCAATAAACGGTGGTGGCGGTAGCGTGGATA
PG40	CTTCGCTGATCAGTTTCTGTTCACCGATATGGGCGTCGCCTTTTGTGG

Table S6: Sequences of templates and primer used for RCA and qPCR

	**Sequence 5' → 3'**
RCA template C	[Phosphate]-TTCCCGGCGGCGCAGCAGTTAGATGCTGCTGCAGCGATACGCGTATCGCTATGGGTAACCGTACGGTTACCCGCAGCAGCATCTAACCGTACAGTATT
RCA Primer C	TCTAACTGCTGCGCCGCCGGGAAAATACTGTACGGTTAGA
Primer qPCR fw	GATGCTGCTGCGGGTAACCG
Primer qPCR rev	CCGGCGGCGCAGCAGTTAG
TaqMan probe	GATACGCGTATCGCTGCAGCAG

Additional References

53. Gibson, D. G.; Young, L.; Chuang, R. Y.; Venter, J. C.; Hutchison, C. A.; Smith, H. O., *Nat. Methods* **2009**, *6*, 343.
54. Bursac, T.; Gralnick, J. A.; Gescher, J., *Biotechnol. Bioeng.* **2017**, *114*, 1283.
55. Maier, M.; Radtke, C. P.; Hubbuch, J.; Niemeyer, C. M.; Rabe, K. S., *Angew. Chem. Int. Ed.* **2018**, *57*, 5539.
56. Yao, C.; Zhang, R.; Tang, J.; Yang, D., *Nat. Protoc.* **2021**, *16*, 5460.
57. Yoshimura, T.; Suzuki, T.; Mineki, S.; Ohuchi, S., *PLoS One*, **2015**, *10* (9), e0136532.
58. Zuker, M., *Nucleic Acids Res.* **2003**, *31*, 3406.
